# Potent Cross-Serotype Dengue Neutralization with Low ADE by AG-Strand-Targeting Anti-EDIII Antibodies

**DOI:** 10.1021/acsomega.6c02114

**Published:** 2026-08-03

**Authors:** Napon Nilchan, Anunyaporn Phungsom, Mongkhonphan Tantiwatcharakunthon, Prasit Luangaram, Pattarakul Pakchotanon, Thaneeya Duangchinda, Romchat Kraivong, Tanapan Prommool, Chatchawan Srisawat, Chunya Puttikhunt

**Affiliations:** † Molecular Biology of Dengue and Flaviviruses Research Team, Medical Molecular Biotechnology Research Group, National Center for Genetic Engineering and Biotechnology (BIOTEC), 67960 National Science and Technology Development Agency (NSTDA), Khlong Nueng, Pathum Thani 12120, Thailand; ‡ Siriraj Center of Research Excellence in Dengue and Emerging Pathogens, Faculty of Medicine Siriraj Hospital, 65106 Mahidol University, Bangkok 10700, Thailand; § Department of Biochemistry, Faculty of Medicine Siriraj Hospital, Mahidol University, Bangkok 10700, Thailand

## Abstract

Dengue virus (DENV) envelope protein domain III (EDIII) is a premier target for vaccine design because EDIII-specific antibodies can potently neutralize DENV with a low propensity for inducing antibody-dependent enhancement (ADE). We previously identified a cross-reactive antibody, R3N_2D3, which targets the EDIII AG-strand epitope and neutralizes all four DENV serotypes with low ADE, a desirable combination of activities among anti-DENV antibodies. In this study, we expanded this AG-strand-targeting antibody panel by identifying four additional anti-EDIII antibodies from our dengue-immune single-chain variable fragment (scFv)-phage libraries. Despite variations in their binding footprints, these new antibodies converged on key residues within the AG-strand epitope. When expressed as full IgG1, these candidates showed potent cross-neutralization with subnanomolar focus reduction neutralization titers (FRNT_50_ values) and consistently low ADE compared to the prototypical fusion-loop antibody 4G2 when tested in an in vitro U937 monocytic cell model. Collectively, these findings demonstrate that the AG-strand epitope can elicit broadly protective antibodies with reduced ADE risk, highlighting its potential as a strategic epitope for pan-DENV vaccine development.

## Graphical abstract

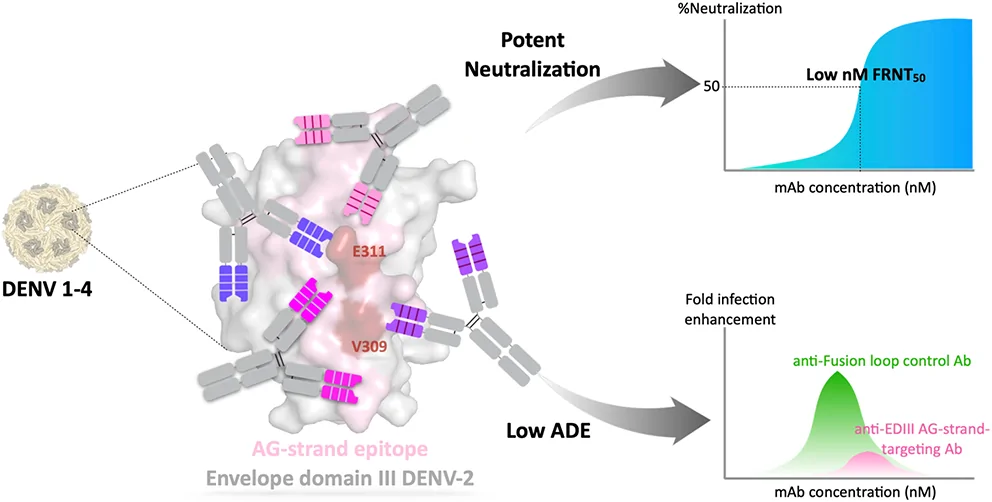

## Introduction

The envelope protein (E) of dengue virus is the primary target of neutralizing antibodies, with neutralization potency heavily dependent on the specific epitopes targeted. While antibodies against the envelope protein domain III (EDIII) constitute only a minor fraction of the total antibody repertoire, monoclonal anti-EDIII antibodies are among the most potent DENV neutralizers identified to date.^1,2^ Furthermore, a subset of anti-EDIII antibodies exhibits low antibody-dependent enhancement (ADE), a phenomenon where subneutralizing antibodies facilitate the infection of Fc-receptor-bearing cells such as monocytes and dendritic cells.^3–6^ Importantly, ADE can contribute to severe disease outcomes, thereby making EDIII an attractive target for developing next-generation dengue vaccines with improved safety profiles and high protective efficacy.^7^

Despite these advantages, the utilization of EDIII in vaccine design presents significant challenges. First, EDIII of certain serotypes can be inherently poorly immunogenic, often requiring intensive immunization strategies to elicit a robust neutralizing antibody response.^8^ Second, EDIII immunization frequently elicits antibodies directed toward the conserved AB-loop epitope, which is poorly neutralizing.^9^ While some of these antibodies have been shown to be low in ADE, such as 2H12 and 3E31, their overall protective efficacy remains limited.^3,4^ Consequently, directing immune responses toward highly neutralizing epitopes of EDIII, such as the lateral ridge (LR) or the AG strand, is a more promising strategy. Eliciting robust responses to these specific sites likely requires the engineering of EDIII immunogens to enhance their immunogenicity while avoiding nonprotective epitopes.^10–12^

The potent neutralizing epitopes of the LR and AG strand partially overlap but are generally associated with distinct immunological activities.^13^ LR-targeting antibodies are generally highly potent and serotype-specific. This type of specificity limits their neutralizing breadth in addition to reducing ADE risk from heterologous serotypes. However, exceptions exist: 2C8, a DENV-2-specific LR antibody, induces substantial ADE, even greater than that caused by antifusion loop epitope 4G2 in in vitro testing.^5,14^ This highlights that ADE from anti-EDIII, while uncommon, can be significant. In contrast, anti-AG-strand antibodies, such as 1A1D-2 and 4E11, tend to be subcomplex reactive, neutralizing multiple, but not all, serotypes. Protein engineering of 4E11 expanded its coverage to include DENV-4 (yielding 4E5A) and eventually the fully humanized variant Ab513/VIS513, which is now in a phase 2 clinical trial.^15–18^ Furthermore, AG-strand antibodies like the germline-like m366.6, derived from a naïve scFv library, achieve cross-serotype neutralization with negligible ADE in DENV-1 and DENV-2, making the AG-strand epitope an especially promising target for the next-generation dengue vaccines.^6^

We previously sought to bias immune recognition toward a potently neutralizing LR epitope recognized by 3H5, a minimal ADE mouse antibody.^14^ Using an engineered EDIII antigen as a “bait”, we selected LR-targeting antibodies, R2N_1G11 and R3N_2D9, which exhibited potent neutralization with minimal ADE against DENV-2. Additionally, two AG-strand-targeting antibodies, R3N_2D3 and R2WT_1B8, were identified. While the discovery of R2WT_1B8 was expected from a control biopanning using wild-type EDIII (EDIII-WT), R3N_2D3 was unexpectedly isolated using an LR-biased antigen (Mut N). Notably, R3N_2D3 was the only antibody that showed broad binding reactivity across all four DENV serotypes. In this study, we evaluated the breadth of neutralization and ADE of R3N_2D3 and R2WT_1B8 with various DENV strains and serotypes. Our findings revealed that R3N_2D3 neutralizes all four DENV serotypes, which prompted us to extend this analysis to a broader set of AG-strand antibodies. We characterized the binding epitopes of these antibodies, their cross-serotype neutralization, and ADE activity. The results demonstrated that AG-strand-targeting antibodies combine broad neutralization with consistently low ADE across all serotypes and thus underscore the advantage of targeting the AG strand as part of a rational EDIII-based dengue vaccine design.

## Results

### R3N_2D3 is a Cross-Serotype Neutralizing Antibody with Low ADE

Two anti-EDIII antibodies targeting the AG-strand epitope, R3N_2D3 and R2WT_1B8, were previously identified and shown to neutralize DENV-2 with minimal ADE.^14^ Given their cross-reactivity with multiple DENV serotypes, we expanded the focus reduction neutralization titer (FRNT) assay to include additional DENV strains and serotypes. The FRNT results revealed distinct differences in neutralization breadth and potency between the two antibodies. Only R3N_2D3 showed neutralization across all tested serotypes and strains, whereas R2WT_1B8 exhibited a more limited neutralization profile (Figure 1A and Supporting Information Figure S1). Despite its high potency against DENV-2, R2WT_1B8 failed to neutralize DENV-3 and displayed varying potency against specific strains of DENV-1 and DENV-4. As anticipated, the LR-targeting antibodies tested in parallel exhibited more type-specific patterns of neutralization: R3N_2D9 neutralized only DENV-2, and R2N_1G11 neutralized both DENV-2 and DENV-4. The observed neutralization breadth is largely consistent with the binding profiles previously reported for R3N_2D3, R2N_1G11, and R3N_2D9. Notably, R2WT_1B8 presented a discrepancy; while it showed binding to DENV-1, DENV-3, and minimally to DENV-4 virions, it only recognized recombinant E proteins of DENV-1 and DENV-2.^14^

**Figure 1:**
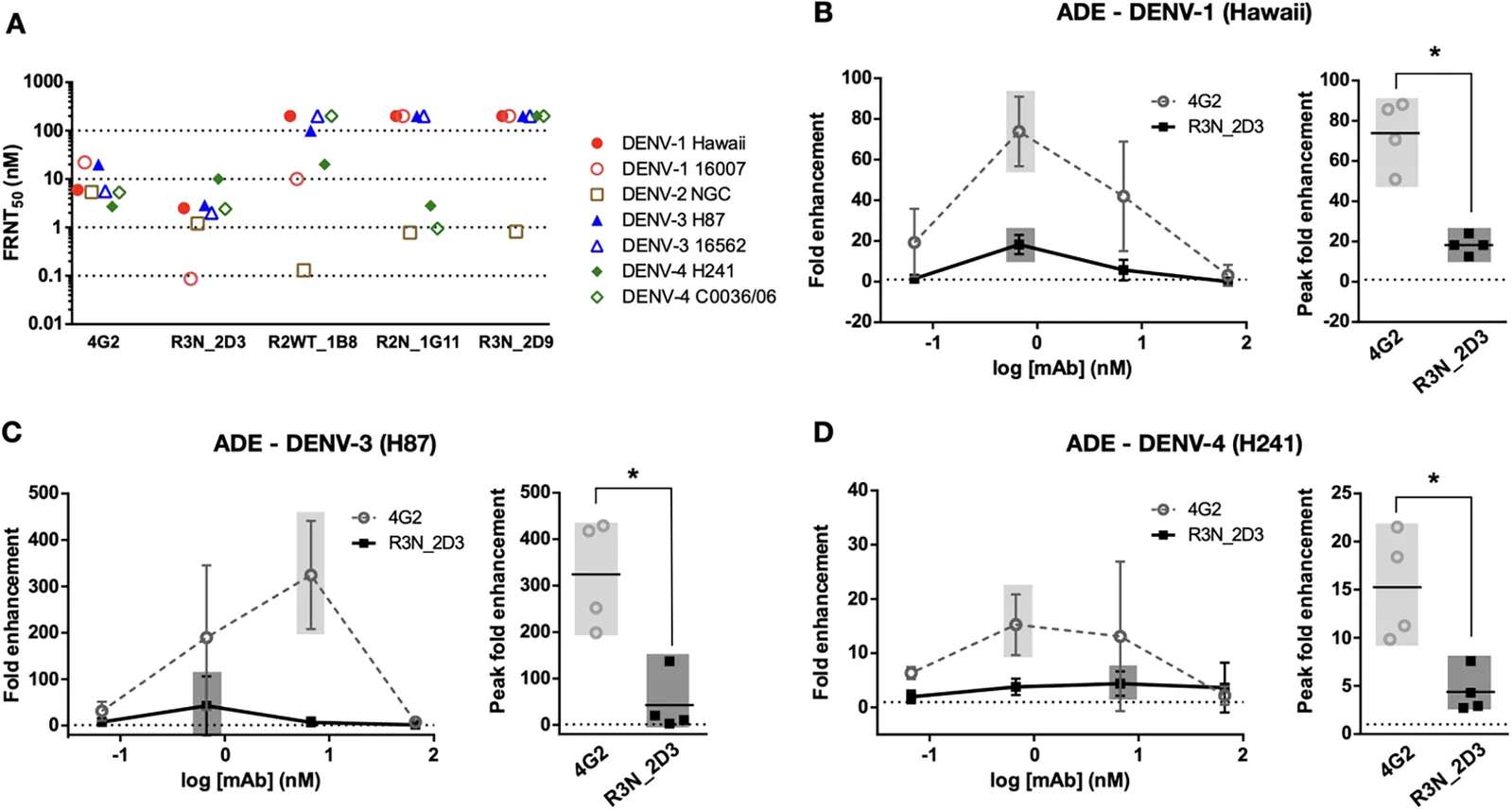
Neutralization and antibody-dependent enhancement (ADE) activities of scFv-phage-derived antibodies. (A) Fifty percent focus reduction neutralization titer (FRNT_50_) values of the antibody panel against different strains and serotypes of DENV. Values represent the averages from two independent experiments. (B–D) ADE assay in U937 monocytic cells against DENV serotypes 1, 3, and 4. Data are from four independent experiments; error bars indicate standard deviation. Individual data points at peak fold enhancement are plotted, with horizontal bars indicating the means. Statistical analysis was performed using an unpaired, nonparametric Mann–Whitney *U* test (*p < 0.05). The horizontal dotted line is a 1-fold enhancement, which represents baseline infection in the absence of an antibody (no enhancement).

We next expand the evaluation of R3N_2D3 to induce in vitro ADE in DENV-1, 3, and 4 with a U937 monocytic cell line model using a fusion-loop-targeting antibody 4G2 as a positive control for a classical enhancement phenotype. Prominent peaks of fold-infection enhancement for 4G2 were observed in DENV-1 (Hawaii) and DENV-3 (H87) infections. Meanwhile, in DENV-4 (H241), the 4G2 peak enhancement was lower than that in other serotypes (∼15-fold) but remained clearly distinguishable from the infection in the absence of an antibody (fold enhancement ≤1, Figure 1B–D). In contrast to 4G2, R3N_2D3 consistently showed lower ADE potential with a lower peak fold enhancement across the three tested serotypes (right panels of Figure 1B–D). The low-level infection enhancement of R3N_2D3 was, however, clearly observed in DENV-1 with an average peak enhancement of ∼18-fold, and in DENV-3, with an average peak enhancement of ∼43-fold. We have also parallel-tested R2N_1G11, R3N_2D9, and R2WT_1B8 for their ADE induction (Supporting Information Figure S2). The LR-targeting R2N_1G11 and R3N_2D9 essentially showed no ADE (fold enhancement ≤ 1), while the subcomplex AG-strand-targeting R2WT_1B8 showed noticeable infection enhancement in DENV-1 and DENV-3 with average peak enhancement of around 33- and 169-fold, respectively. We noted that in the DENV-4 assay, all anti-EDIII antibodies showed peak enhancement of <5-fold. Collectively, these data distinguish the cross-neutralizing, low ADE profile of the AG-strand-targeting R3N_2D3 from a classic cross-reactive and enhancing antibody 4G2. This favorable combination of activities of R3N_2D3 prompted us to assess whether other fully cross-reactive AG-strand antibodies share these protective characteristics.

### Identification of Novel Cross-Reactive AG-Strand-Targeting Antibodies from the scFv-Phage Library

To further investigate the properties of AG-strand-targeting antibodies, we analyzed scFv-phage clones from a biopanning campaign using the wild-type, unmodified EDIII antigen (EDIII-WT). We specifically focused on clones exhibiting reduced binding to the EDIII-309N_311T variant, which carries a glycosylation site at residue 309 designed to sterically shield the AG-strand epitope, a strategy previously validated for antibody 513.^14^ We reasoned that such sensitivity would identify scFv phages targeting this specific site on EDIII. Four distinct scFvs were selected based on sequence identity: R2WT_1C1, R2WT_1C3, R3WT_2B2, and R3WT_2C3 (hereafter referred to as 1C1, 1C3, 2B2, and 2C3, respectively).

Sequence analysis using IMGT/V-QUEST revealed that all four clones utilize the same V_H_ gene but differ in their V_L_ gene combinations. At the amino acid level, the V_L_ sequence showed <85% identity, while the V_H_ sequences remain nearly identical (92–98% identity). (Figure 2A and Supporting Information Figure S3). Notably, these V_H_ sequences are distinct from those of R2WT_1B8 and R3N_2D3 (∼40% identity). We noted that the V_L_ sequence of 2C3 shares 88.9% identity with R2WT_1B8, despite possessing a divergent V_H_ domain (45.3% identity).

**Figure 2:**
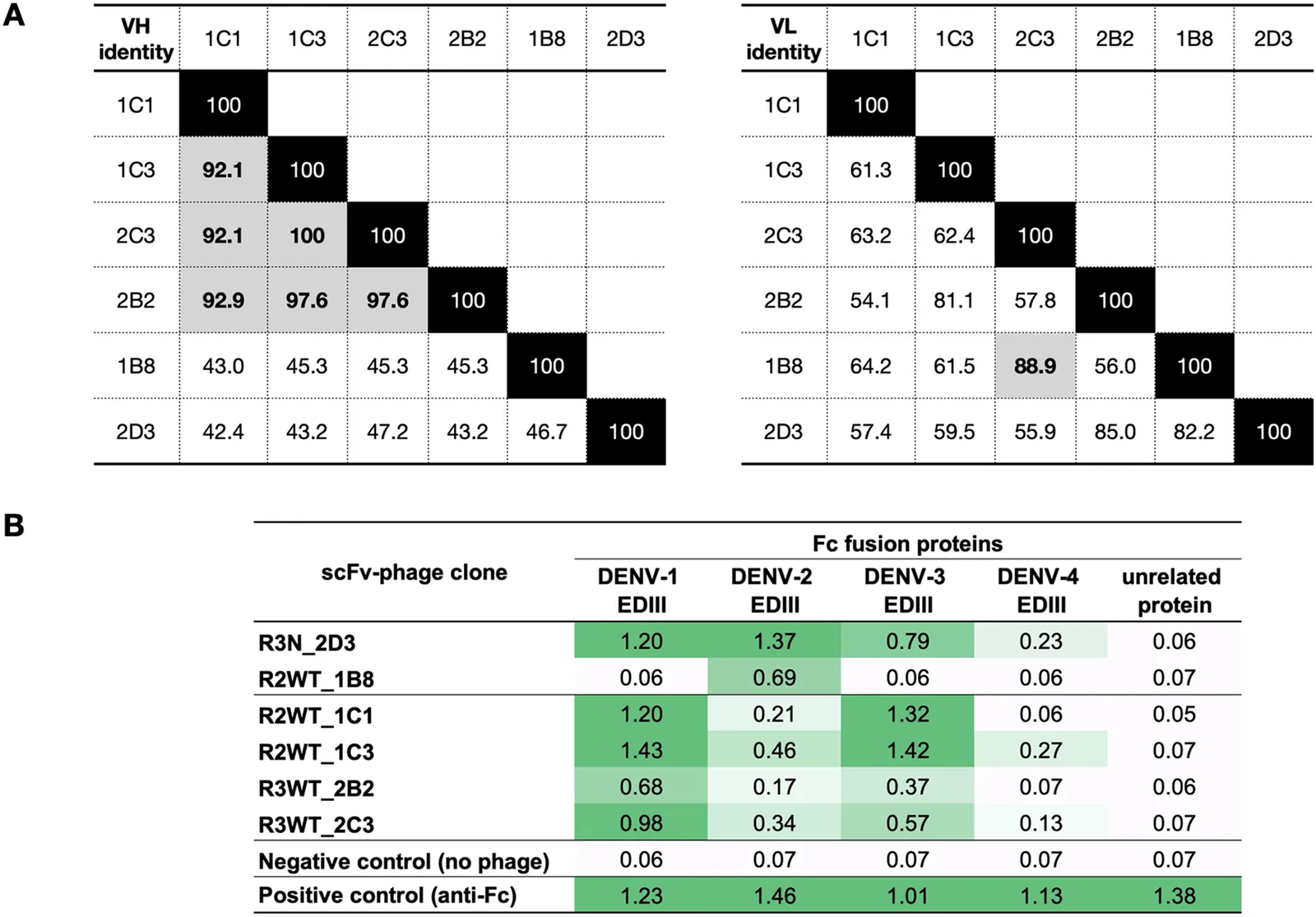
Characterization of selected AG-strand scFv phages. (A) Percentage amino acid identity of V_H_ and V_L_ sequences (abbreviations: 1B8 = R2WT_1B8; 2D3 = R3N_2D3). (B) Cross-reactivity of the selected scFv phages against EDIII-Fc antigens from different DENV serotypes, measured by ELISA. Values represent the average absorbance of two technical replicates.

We first evaluated the cross-reactivity of new scFv-phage clones against a panel of recombinant EDIII-Fc antigens of all four serotypes (Supporting Information Figure S4). The clones demonstrated broad cross-reactivity across 3–4 serotypes, although reactivity against DENV-4 EDIII was consistently weak (Figure 2B). R3N_2D3 and R2WT-1B8 phages were included as controls; while R3N_2D3 maintained cross-serotype reactivity, the R2WT_1B8 phage bound specifically to EDIII of serotype 2. This restricted specificity of R2WT_1B8 scFv phage contrasts with the subcomplex reactivity observed in its full-length IgG1 format, likely reflecting differences in binding avidity or conformational constraints between the two formats.

To define the binding epitopes, we performed ELISA using a panel of DENV-2 EDIII (D2EDIII-Fc) mutants. Binding residues were identified by reduction in reactivity relative to EDIII-WT: “key residues” were defined by >75% reduction in binding (<25% relative binding), while “peripheral residues” showed 25–75% relative binding. Although the four new clones displayed distinct sets of binding residues, their footprints converged on residues V309 and E311; mutations at these positions severely abrogate binding of all four clones (Figure 3A). In contrast, R3N_2D3 and R2WT_1B8 were unaffected by the mutations at E311 and only moderately sensitive to glycosylation at V309. Furthermore, both R2WT_1B8 and R3N_2D3 were sensitive to mutations at position 305, a residue critical for several LR-targeting antibodies, including 3H5, Z004, Z006, and E16.^5,19,20^ Among the newly identified clones, only 2B2 was moderately affected by the glycosylation at position 305 (K305N) (Figure 3B).

**Figure 3:**
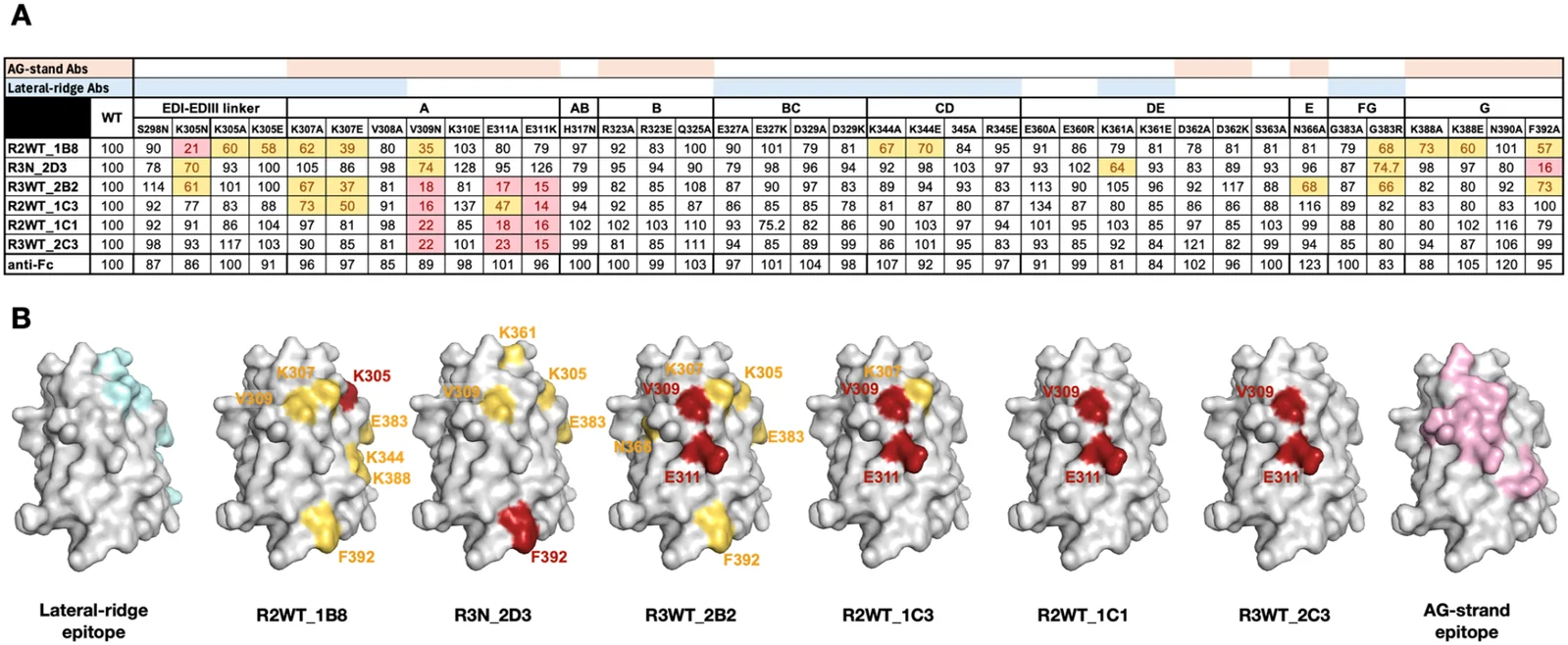
Epitope mapping of the scFv phage using capture ELISA. (A) Epitope mapping of the scFv phage with a panel of D2EDIII-Fc mutants. Key binding residues (red) correspond to mutations that reduce binding to less than 25% relative to the wild-type D2EDIII-Fc (WT). Peripheral binding residues (yellow) correspond to mutations that reduce binding to 25–75% relative to WT. Epitope residues of AG-strand antibodies (513, 1A1D-2, 4E5A) and lateral-ridge Abs (3H5, 2C8, 9F16) are shown for comparison. (B) Illustrations of key and peripheral binding residues of each scFv phage on a surface of a three-dimensional structure of DENV-2 EDIII (extracted from the crystal structure of DENV-2 envelope protein, PDB ID:1OAN). The structural components of EDIII were represented as A = A strand; AB = AB loop; B = B strand; BC = BC loop; CD = CD loop; DE = DE loop; E = E strand; FG = FG loop; and G = G strand.

### AG-Strand-Targeting Antibodies Exhibit Potent Neutralization with Low ADE

To evaluate the neutralization potency of the newly identified AG-strand binders, the V_H_ and V_L_ genes were cloned into a full human IgG1 framework. Following expression and purification, the reactivity and specificity of these IgG1 antibodies to DENV envelope protein was validated by ELISA and Western blot (Figure 4A–D and Supporting Information Figure S5). The ELISA results confirmed that these antibodies are cross-reactive, with midpoint binding half-maximal effective concentration (EC_50_) in the subnanomolar range across four serotypes. We also noted that the binding to DENV-4 EDIII-Fc antigen that appeared weak in the monovalent scFv-phage format has become apparent, possibly due to the avidity effect inherent to the bivalent architecture of IgG. The binding kinetics to soluble envelope proteins (E80) were measured using surface plasmon resonance (SPR). The SPR results in Figure 4E and Supporting Information Figure S6 show that these antibodies exhibit exceptionally high on-rate (*k*_a_ ∼ 10^5^–10^6^ M^–1^s^–1^) across all serotypes. Additionally, they possess high affinities toward E80 of DENV-1, DENV-2, and DENV-3 with dissociation constants (K_D_) ranging from 0.017 to 1.30 nM, while binding to DENV-4 E80 appeared to be around an order of magnitude lower in affinity, yielding *K*_D_ values ranging from 0.620 to 10.4 nM. This reduced affinity toward DENV-4 E80 is driven primarily by a faster off-rate (*k*_d_) of ∼10^–2^ to 10^–3^s^–1^ compared to *k*_d_ of 10^–4^ to 10^–5^ s^–1^ in other serotypes.

**Figure 4:**
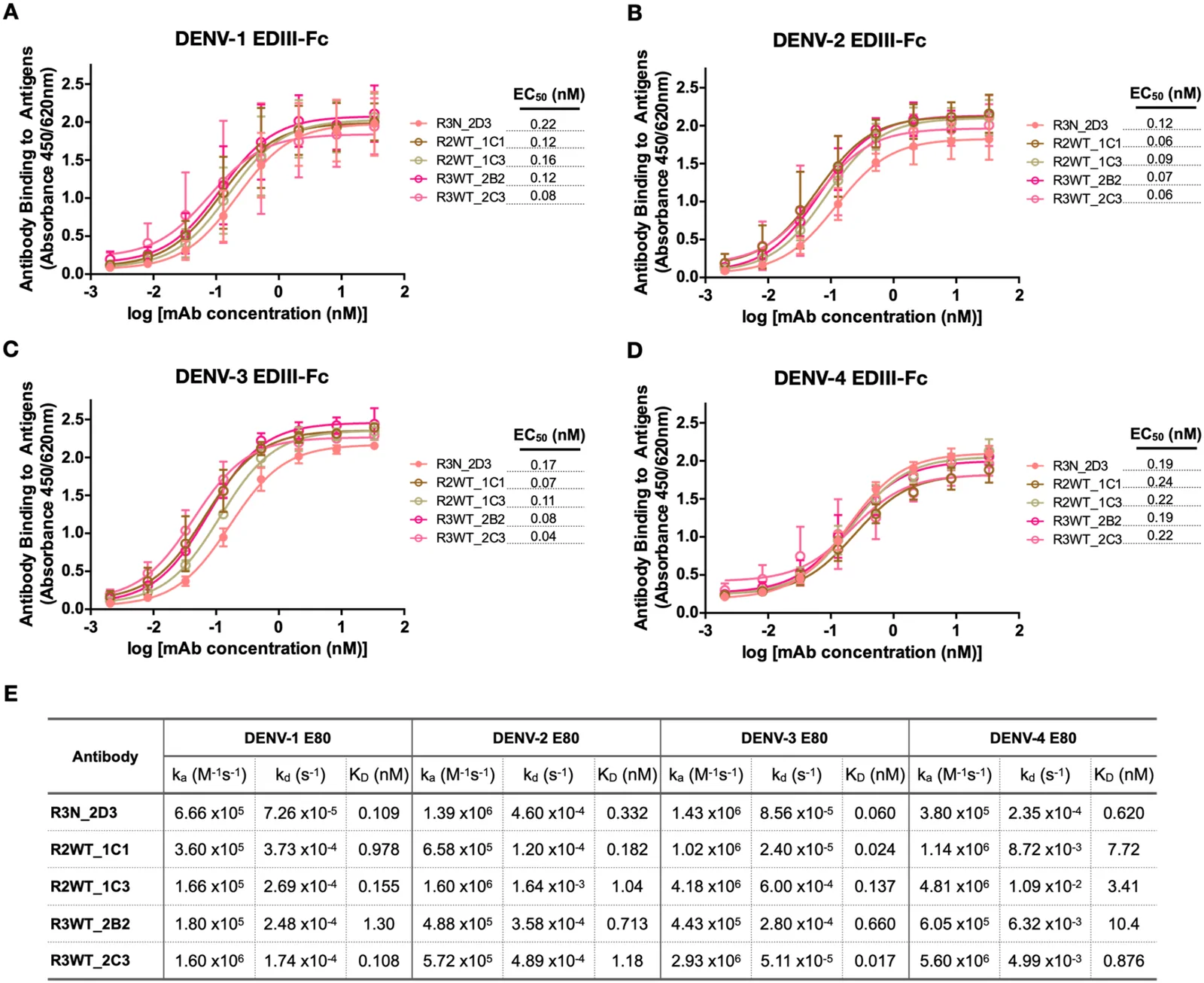
Cross-reactivity of the scFv-derived AG-strand antibody panel. (A–D) ELISA against recombinant EDIII-Fc antigens. Three independent experiments were performed; error bars indicate standard deviation. (E) Binding kinetics to recombinant soluble envelope protein E80 (DENV E80) from surface plasmon resonance measurement.

We subsequently characterized the virus-neutralization activity of these antibodies. All four antibodies demonstrated potent neutralization against all four DENV serotypes, with 50% focus reduction neutralization titer (FRNT_50_) values generally ranging from subnanomolar to a single-digit nanomolar scale, except 2C3, which exhibited a higher FRNT_50_ in a double-digit nanomolar concentration against DENV-4 (Figure 5A and Supporting Information Figure S7).

**Figure 5:**
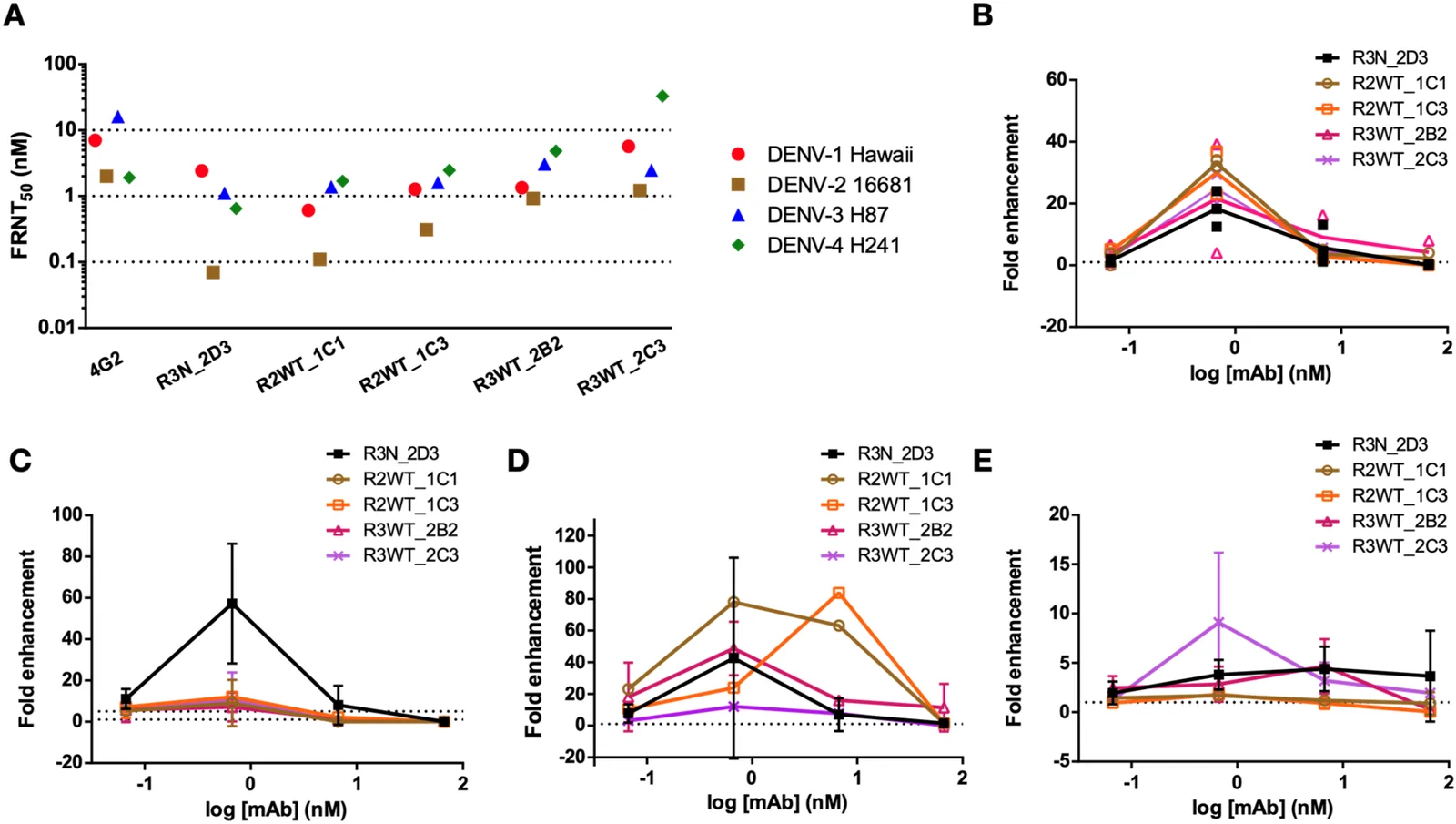
Neutralization and ADE activities of scFv-phage-derived antibodies. (A) FRNT_50_ values of the antibody panel against four serotypes of DENV. Values are the averages of two independent experiments. (B–E) ADE assay in the U937 monocytic cell line against DENV serotypes 1, 3, and 4. Two independent experiments were performed; error bars indicate standard deviation. The horizontal dotted line is a 1-fold enhancement, which represents baseline infection in the absence of antibody (no enhancement).

In vitro ADE assays further revealed that all four new antibodies exhibit generally similar infection-enhancement profiles as compared to R3N_2D3 (Figure 5B–E), especially in DENV-1. In DENV-2, the four new antibodies appeared to show lower peak enhancement than that caused by R3N_2D3, while in DENV-4, 2C3 is the only clone that showed a tendency of higher ADE peak with an average ∼9-fold enhancement, while other clones exhibited <5-fold enhancement. For DENV-3, the ADE enhancement profile of the reference R3N_2D3 exhibited experimental variability at its peak concentration (∼0 log­[mAb] (nM)), resulting in a wide error bar that precludes a comparison between individual clones. Nevertheless, the peak fold enhancement of all four newly identified clones fell within the dynamic error range of R3N_2D3. While the assay variability limits our ability to resolve the differences between these antibodies, their overall profiles collectively cluster with the same enhancement window as the reference antibody. Collectively, these data suggest that the AG-strand epitope of EDIII elicits antibodies that combine broad neutralization breadth with a low ADE profile, similar to what was observed in R3N_2D3.

## Discussion

In this study, we characterized a panel of anti-EDIII monoclonal antibodies that recognize the AG-strand epitope and assessed both their neutralization breadth and ADE potential across four serotypes. Our results demonstrate that AG-strand-targeting antibodies can achieve broad reactivity and potent neutralization with consistently low ADE, as exemplified by R3N_2D3. In contrast, R2WT_1B8 showed restricted binding and neutralization that was specific to certain serotypes or strains. This narrower reactivity was accompanied by more pronounced ADE activities in DENV-1 (Hawaii) and DENV-3 (H87). The observed functional divergence underscores that AG-strand antibodies are not monolithics; rather, epitope fine specificity influences protective efficacy. Nevertheless, the ability of R3N_2D3 to combine broad neutralization with low ADE highlights the AG-strand epitope as a strategic target for rational vaccine design.

Expanding our analysis to four additional phage-derived AG-strand antibodies (1C1, 1C3, 2B2, and 2C3) revealed that these new antibodies exhibited full cross-neutralization and low ADE, mirroring those of R3N_2D3. Unlike R3N_2D3 and R2WT_1B8, the binding footprints of these four clones converged on residues V309 and E311. Notably, their activity profiles resemble that of the germline-like antibody m366.6, one of the few reported anti-EDIII antibodies capable of cross-neutralizing DENV with an undetectable ADE. While 1C1, 1C3, 2B2, and 2C3 share highly similar V_H_ sequences, their diverse V_L_ domains did not appear to significantly impact cross-reactivity or ADE profiles. Conversely, although 2C3 and R2WT_1B8 share 88.9% identity V_L_, they possess different V_H_ domains, resulting in vastly different reactivities toward recombinant EDIII antigens. We hypothesize that the V_H_ domain may play a dominant role in conferring cross-reactivity. Given the noncognate nature of V_H_–V_L_ pairing within the scFv-phage library, this hypothesis warrants future structural characterization or further domain-swapping validation.

The V_H_ of 1C1, 1C3, 2B2, and 2C3 use the same IGHV1–18 gene segment that was previously suggested to be part of the ‘innate-like’ antiviral recognition system, production of polyspecific antibodies, and potentially contribute to ADE.^22^ Our small set of these IGHV1–18-encoded antibodies suggests that these antibodies exhibit specific binding and high affinity to dengue envelope proteins and lower ADE potential compared to a more well-known ADE-inducing antibody 4G2. Intriguingly, sequence analysis also showed that R3N_2D3 utilizes the IGHV3–23 heavy chain variable gene segment, a germline used among recurrent potent human antilateral ridge (LR) antibodies of ZIKV EDIII.^19,23^ While those anti-ZIKV antibodies are restricted to an IGHV3–23/IGKV1–5 pairing, all other scFv clones clonally related to R3N_2D3 previously identified exclusively utilized an IGHV3–23/IGLV3–21 pairing.^14^ This may indicate a highly stringent structural requirement for the IGHV3–23 to effectively target DENV or ZIKV EDIII. This strict pairing is in sharp contrast to the IGHV1–18-encoded clones (1C1, 1C3, 2B2, and 2C3), which maintain their broad neutralization breadth across more diverse light-chain partners.

Our mapping experiment also indicated that the binding of R3N_2D3, R2WT_1B8, and 2B2 is sensitive to mutations at positions 305 (A-strand) and 383 (FG loop), residues typically associated with LR-antibody recognition. This suggests that their epitopes may be slightly shifted toward the LR region, whereas 1C1, 1C3, and 2C3 are more centrally focused on residues 309 and 311 of the A-strand. These observations emphasize that the AG-strand and LR epitopes exist as a continuous binding landscape targeted by antibodies with a spectrum of protective properties and reactivity.

Potent neutralizing antibodies with low or no ADE were once regarded as uncommon in dengue research. However, a growing body of research, including our findings, suggests that such antibodies, particularly those targeting EDIII, might be more prevalent than previously recognized. For example, 3H5 was described as a highly unusual minimally enhancing antibody. Similarly, 2H12 and 3E31, which target a moderately to low neutralizing AB-loop epitope, were also shown to have low to no ADE, as did AG-strand antibodies such as m366.6, R3N_2D3, and four clones reported here. Although Fc engineering could effectively eliminate ADE by disrupting Fc-receptor interactions, steering the immune response toward potent neutralizing epitopes with naturally lower ADE propensity, such as AG-strand and LR epitopes of EDIII, may provide an additional strategy for effective dengue vaccine design.

## Limitations and Future Directions

There are several limitations to our work that warrant consideration. First, we examined a relatively small set of AG-strand antibodies, most of which use similar V_H_ gene segments. While this sequence convergence implies a shared structural basis for the observed functionalities tested, future studies with larger and more diverse repertoires are necessary to establish the generality of these findings. Additionally, in vivo protection studies and high-resolution structural analysis (e.g., cryo-EM or X-ray crystallography) will be essential to clarify the precise mechanisms of these cross-serotype neutralization and antibody-dependent enhancement (ADE) minimization. Second, ADE is a well-documented, cell-type-dependent phenomenon. Although our in vitro U937 monocytic model could provide a baseline to compare the enhancement magnitude across different antibody clones, expanding these evaluations to broader cell types (such as K562 or primary myeloid cells) and animal models is required to confirm the physiological relevance of the observations. Third, our mutant antigen panel provided a practical and efficient strategy to map the binding footprint under resource-limited settings, but it may not fully capture the complexity of antibody fine specificity on actual virus particles. Finally, our antibodies were generated through an scFv-phage library approach with inherent noncognate V_H_–V_L_ pairing. However, the identification of multiple clones with similar V_H_ or V_L_ sequences and functions could ascertain that these gene families are relevant to the in vivo response.

## Conclusions

Taken together, our findings support the AG-strand epitope as a promising target for DENV vaccine design. Antibodies directed to this epitope can achieve broad, potent neutralization while exhibiting a low ADE profile. We propose that the AG-strand and LR epitopes represent a landscape of binding regions that can be strategically targeted to combine the type-specific potency of LR-directed antibodies with the broad-serotype breadth of AG-strand-directed antibodies. Our data reinforce the notion that low ADE is a general feature of anti-EDIII antibodies, with rare exceptions such as 2C8. Rational engineering of EDIII to focus the immune response toward these protective “safe zones”, while masking ADE-prone regions or poorly neutralizing epitopes like the AB loop, offers a viable pathway for the development of effective subunit vaccines for dengue and related flaviviruses.

## Methods

### Cell Lines and Virus

Vero cells (African green monkey kidney cell line) were cultured in MEM; U937 (human monocytic cell line) in RPMI 1640 medium; HEK293T (human embryonic kidney cell line) in DMEM. All media were supplemented with 10% heat-inactivated fetal bovine serum (FBS), 1x penicillin-G/streptomycin, and 2 mM l-glutamine. Dengue virus serotype-1 (DENV-1, Hawaii and 16007), DENV-2 (16681 and NGC), DENV-3 (H87 and 16562), and DENV-4 (H241 and C0036/06) were propagated in mosquito cell line C6/36, which was cultured in Leibovitz-15 medium supplemented with 3% heat-inactivated FBS and 10% tryptose phosphate broth. scFv phages were generated from single colonies of *E. coli* XL-1 Blue infected with helper phage M13KO7 (NEB) and cultured in 2xYT medium supplemented with 0.1% glucose, 50 μg/mL ampicillin, and 50 μg/mL kanamycin. The overnight culture containing scFv phage was clarified and used for experiments.

### Cloning and Protein Expression

#### Antigens

EDIII-Fc antigens were cloned, expressed, and purified as previously described.^14^ Briefly, EDIII genes of DENV-1 Hawaii, DENV-2 16681, DENV-3 H87, and DENV-4 H241 were amplified from pMT-D1E80, pMT-D2E80, pMT-D3E80, and pMT-D4E80 (unpublished) and cloned into an expression vector containing Fc of a human IgG1 antibody and a hexa-histidine tag at the C-terminus of the Fc. Mutants of the DENV-2 EDIII-Fc antigen (D2EDIII-Fc) were generated from standard PCR mutagenesis. All constructs were transiently expressed in HEK293T cells using linear PEI (25 kDa, Polysciences) and purified with a cobalt-immobilized resin column (TALON).

#### Antibodies

This study used a previously constructed human antibody phage display library derived from peripheral blood mononuclear cells (PBMCs), which was generated and reported previously.^14,21^ The library was established under the approval of the Siriraj Institutional Review Board (protocol number 632/2559), Khon Kean Hospital Institutional Review Board in Human Research (protocol number KE60108), and the Ethical Committee of Songkhla Hospital (protocol number 11/256) with informed consent from all donors. No new human samples or data were collected in the present study.

scFv-derived antibodies were cloned by amplifying the V_H_ and V_L_ genes from the phagemids and inserting the amplicons into vectors containing the constant regions of the human IgG1 heavy chain or the constant region of the human λ light chain. The constructs were transiently expressed in HEK293T cells and purified with protein A agarose beads (Sigma-Aldrich). Purity was confirmed by Coomassie-stained SDS-PAGE, and the concentration was determined by UV absorption at 280 nm (NanoDrop).

### ELISA

Maxisorp plates (Nunc) were coated with antigens or capture antibodies. Plates were subsequently washed with PBS and blocked with appropriate blocking solutions at 37 °C for 1 h. After blocking, primary antibodies or phage culture was added and incubated at 37 °C for 1 h. Subsequently, plates were washed and incubated with appropriate secondary antibodies at 37 °C for 1 h, washed, and developed with Ultra 1-Step TMB (Thermo Scientific). TMB signal was quenched with 2 M H_2_SO_4_ and immediately measured the absorbance at 450/620 nm (TECAN Sunrise).

#### Phage Indirect ELISA

Plates were coated with 1 μg/mL of EDIII-Fc or an unrelated Fc-fusion protein. A solution of 2% skim milk in PBS was used as a blocking solution and diluent. Phage culture was diluted at a 2:3 ratio and used as a primary antibody. An anti-M13-HRP antibody (GE Healthcare) at a 1:2,500 dilution was used as a secondary antibody. An anti-Fc antibody-HRP (AP112P, goat anti-human IgG H+L, Merck) was used as a positive control for all Fc antigens.

#### Phage-Capture ELISA for Epitope Residue Mapping

Plates were coated with a rabbit anti-human γ-chain-specific antibody (A0423, Dako) at 1:2500 dilution. Subsequently, plates were washed, blocked with 2% skim milk, and incubated with 200 ng/mL of DENV-2 EDIII-Fc mutant antigens. Subsequently, the bound antigens were detected with a diluted scFv-phage culture. An anti-M13-HRP antibody at a 1:2,500 dilution (GE Healthcare) was used as a secondary antibody. To ensure comparable antigens, a control experiment was done in parallel using a rabbit anti-human γ-chain-specific antibody (P0214, Dako, 1:5,000 dilution).

#### Antibody ELISA

Plates were coated with 1 μg/mL of EDIII-Fc or an unrelated Fc-fusion protein. A 1% bovine serum albumin (BSA) solution was used as a blocking solution and diluent. Primary antibodies were diluted to 5 μg/mL in diluent. A goat anti-human Ig lambda light-chain-HRP (A506P, Merck) was used at 1:4000 as a secondary antibody.

##### Surface Plasmon Resonance (SPR)

Binding affinities of the antibodies were measured by SPR using a Biacore X100 instrument (GE Healthcare). The single-cycle kinetic (SCK) method was employed, as our initial trial run indicated that our antibody panel exhibits rather tight binding, with the dissociation rate (*k*_d_) approaching the limit of the instrument in certain cases. Briefly, an antibody was immobilized onto a sensor chip protein-G (Cytiva) at approximately 100–200 RU. Varying concentrations of DENV-1 to -4 soluble envelope protein (E80) were injected at a flow rate of 30 μL/min using the TNE buffer pH 7.4 supplemented with 0.01% Tween-20. For clones that the SCK method gave suboptimal results, a standard multiple-cycle kinetic (MCK) method was performed. Both methods were run using an identical capture architecture to ensure direct comparability of the calculated parameters. The association and dissociation data were fitted with Biacore X100 software with a 1:1 interaction model.

##### Western Blots

C6/36 infected with DENV-1 (Hawaii), DENV-2 (16681), DENV-3 (H87), DENV-4 (H241), or mock were lysed in the RIPA buffer. Lysates were resolved by SDS-PAGE. The proteins were subsequently transferred onto nitrocellulose membranes, blocked with 5% skim milk, and cut into strips. The strips were probed with anti-EDIII antibodies or an anti-E chimeric 4G2 (ch4G2) as a control, followed by a goat anti-human IgG (H+L)-HRP (AP112P, Merck) at 1:5,000 dilution in 5% skim milk. The signal was then developed with a LumiFlash Prime ECL substrate (Visual Protein) and imaged with ImageQuant 800 (Cytiva).

##### Focus Reduction Neutralization Test (FRNT) and Antibody-Dependent Enhancement (ADE)

The FRNT and the ADE assay were adapted from.^5^ For FRNT, a serial dilution of a testing antibody was incubated with a ∼10^2^ foci-forming unit (FFU) of DENV at 37 °C for 1 h. The virus–antibody mixture was then transferred to Vero cells, incubated to infect the cells at 37 °C for 2 h, then overlaid with 1.3% carboxymethylcellulose in a 2% FBS MEM. The assay was incubated for 48–72 h, after which cells were fixed, permeabilized, and stained with a mouse anti-E clone 4G2, followed by an antimouse IgG-HRP (AP124P, Merck, at 1:750 dilution or P0260, Dako, at 1:2,000 dilution). Foci were then stained with DAB substrate and visualized under BioSpot (CTL technologies). Foci numbers were enumerated, and the neutralization curves were fitted using a 3-parameter nonlinear regression analysis (GraphPad Prism).

For the ADE assay, serial dilutions of antibodies were incubated with DENV at a multiplicity of infection (MOI) of 0.5 at 37 °C for 1 h. The virus–antibody mixture was then transferred to U937 cell culture and incubated for 72–96 h, after which culture supernatant was harvested and titrated on monolayered Vero cells as in FRNT. Fold of infection enhancement (fold enhancement) was calculated from the number of foci in the presence of antibody divided by the number of foci in the absence of antibody. The obtained fold enhancement was plotted against antibody dilution using GraphPad Prism.

## Supplementary Material



## Notes

Focus reduction neutralization titer plots and FRNT_50_ values of the initial panel scFv-derived antibodies; an immunoglobulin gene analysis of scFv-derived antibodies in this study; Coomassie-stained SDS-PAGE analysis of recombinantly expressed EDIII-Fc antigens and an unrelated Fc-fusion protein (scFvFc) used in ELISA; confirmation of DENV envelope protein specificity of antibodies in this study with Western blot analysis of DENV-infected C3/36 cell lysates; confirmation of DENV envelope (E) protein specificity of antibodies in this study with Western blot analysis of DENV-infected C3/36 cell lysates; surface plasmon resonance sensograms of antibodies in this study; and focus reduction neutralization titer plots and FRNT_50_ values of cross-reactive AG-strand-targeting scFv-derived antibodies in this study (PDF)
