# Supplementary material for: Potent Cross-Serotype Dengue Neutralization with Low ADE by AG-Strand-Targeting Anti-EDIII Antibodies
Source: ACS Omega. 2026 Aug 3;11(32):47592–601. doi: 10.1021/acsomega.6c02114 (PMC13491982; doi:10.1021/acsomega.6c02114)
Supplement: Supplementary file 1 [file ao6c02114_si_001.pdf]

Supporting Information

Potent Cross-Serotype Dengue Neutralization with Low ADE by AG-Strand-Targeting Anti-EDIII Antibodies

Napon Nilchan[1,2], Anunyaporn Phungsom[1,2], Mongkhonphan Tantiwatcharakunthon[1,2], Prasit Luangaram[1,2], Pattarakul Pakchotanon[1,2], Thaneeya Duangchinda[1,2], Romchat Kraivong[1,2], Tanapan Prommool[1,2], Chatchawan Srisawat[3], Chunya Puttikhunt[1,2]**

[1]Molecular Biology of Dengue and Flaviviruses Research Team, Medical Molecular Biotechnology Research Group, National Center for Genetic Engineering and Biotechnology (BIOTEC), National Science and Technology Development Agency (NSTDA), Pathum Thani 12120, Thailand.

[2]Siriraj Center of Research Excellence in Dengue and Emerging Pathogens, Faculty of Medicine Siriraj Hospital, Mahidol University, Bangkok 10700, Thailand.

[3]Department of Biochemistry, Faculty of Medicine Siriraj Hospital, Mahidol University, Bangkok 10700, Thailand.

*Corresponding authors: napon.nil@biotec.or.th and chunyapk@hotmail.com

TABLE OF CONTENTS

	Page
**Supplementary Figure S1**<br>Focus reduction neutralization titer plots and $FRNT_{50}$ values of the initial panel scFv-derived antibodies.	S3
**Supplementary Figure S2**<br>ADE assay of R2WT_1B8, R2N_1G11 and R3N_2D9 in U937 monocytic cell line.	S3
**Supplementary Figure S3**<br>An immunoglobulin gene analysis of scFv-derived antibodies in this study.	S4
**Supplementary Figure S4**<br>Coomassie-stained SDS-PAGE analysis of recombinantly expressed EDIII-Fc antigens and an unrelated Fc-fusion protein (scFvFc) used in ELISA.	S4
**Supplementary Figure S5**<br>Confirmation of DENV envelope (E) protein specificity of antibodies in this study with western blot analysis of DENV-infected C3/36 cell lysates.	S5
**Supplementary Figure S6**<br>Surface plasmon resonance sensograms of antibodies in this study	S6-S11
**Supplementary Figure S7**<br>Focus reduction neutralization titer plots and $FRNT_{50}$ values of cross-reactive AG-strand targeting scFv-derived antibodies in this study.	S12

Supplementary Figure S1

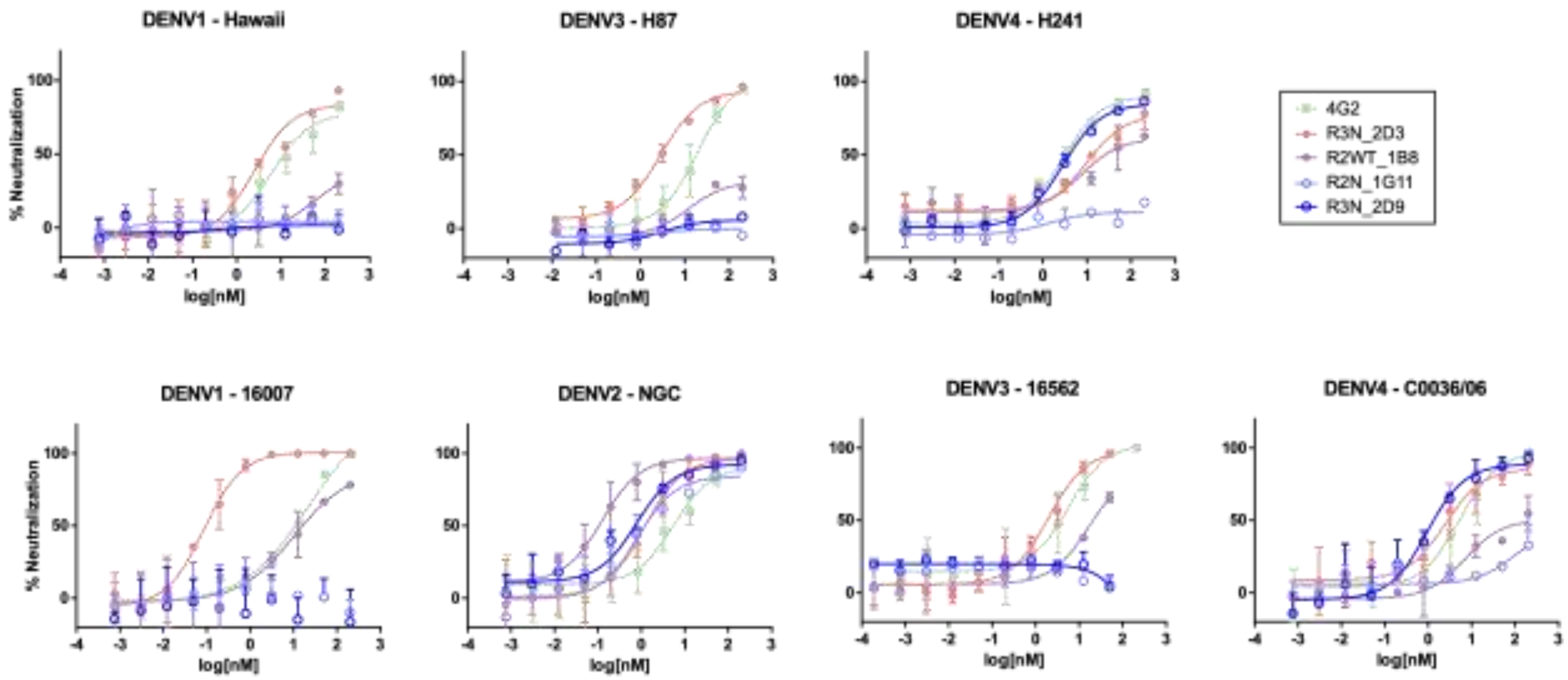

Virus		FRNT50 (nM)					Sources
		Anti-EDIII				Anti-FLE	
Serotype	strain	R3N_2D9	R2N_1G11	R2WT_1B8	R3N_2D3	4G2	
DENV-1	Hawaii	>200	>200	>200	**2.5**	**6.0**	This work
	16007	>200	>200	**10**	**0.087**	**22**	This work
DENV-2	16681	**0.12**	**0.047**	**0.008**	**0.12**	**0.54**	Nilchan et al., 2024
	NGC	**0.82**	**0.78**	**0.13**	**1.2**	**5.4**	This work
DENV-3	H87	>200	>200	>200	**2.9**	**20**	This work
	16562	>200	>200	>100	**2.0**	**5.6**	This work
DENV-4	H241	>200	**2.8**	**20**	**10**	**2.7**	This work
	C0036/06	>200	**0.95**	>200	**2.4**	**5.3**	This work

Supplementary Figure S1. Focus reduction neutralization titer plots and $FRNT_{50}$ values of the initial panel scFv-derived antibodies (both LR-specific and AG-strand specific).

Supplementary Figure S2

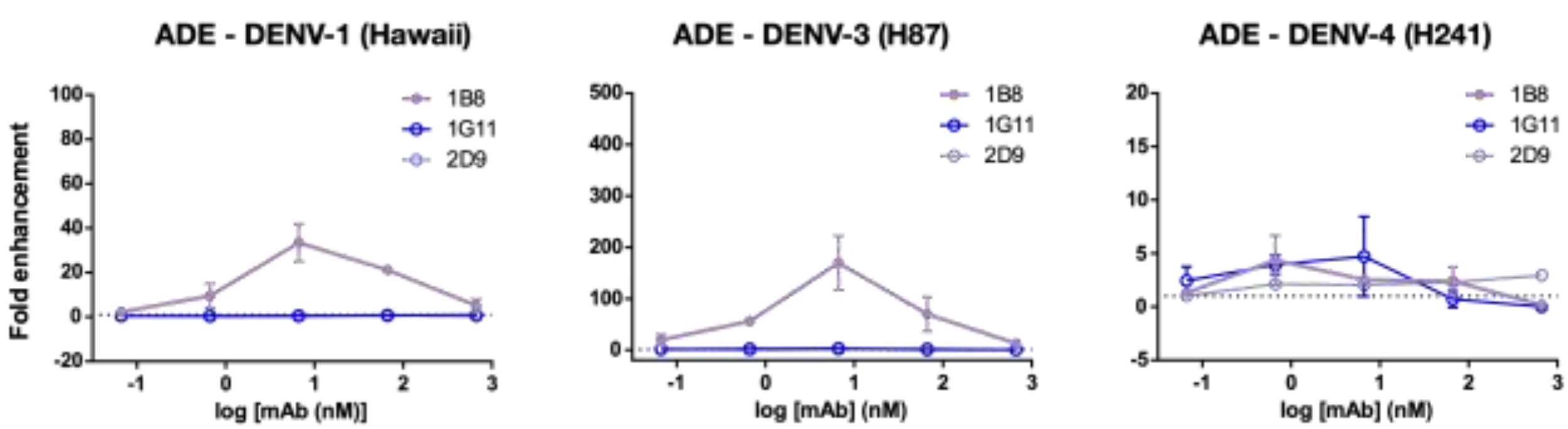

Supplementary Figure S2. ADE assay of R2WT_1B8, R2N_1G11 and R3N_2D9 in U937 monocytic cell line. Data presented are from two independent experiments; error bars indicate standard deviation. The horizontal dotted line is 1-fold enhancement which represents baseline infection in the absence of antibody (no enhancement).

Supplementary Figure S3

Clone	IGH germline genes			HCDR3 amino acids	IGL germline genes		LCDR3 amino acids	Sources
	H - V gene	H - D gene	H - J gene		L - V gene	L - J gene		
R3N_2D3	V3-23*04 F	D6-6*01 F	J4*02 F	CAKGRYASPSPPFDPW	V3-21*04 F	J2*01 F, or J3*01 F or J3*02 F	CQVWDSGNDSLGGS	Nilchan et al., 2024
R2WT_1B8	V4-39*01 F	D6-19*01 F	J4*02 F	CARQAQWMAMAFDHW	V3-21*04 F	J2*01 F, or J3*01 F	CQVWDSGRDHVLF	Nilchan et al., 2024
R3WT_2C3	V1-18*01 F	D6-19*01 F	J1*01 F	CARVQFYWSGSDLRVVGFQHW	V3-21*04 F	J2*01 F, or J3*01 F	CQVWDSSSDHVVF	This work
R2WT_1C3	V1-18*01 F	D6-19*01 F	J1*01 F	CARVQFYWSGSDLRVVGFQHW	V1-40*01 F	J2*01 F, or J3*01 F or J3*02 F	CQSYDSSLSGVF	This work
R2WT_1C1	V1-18*01 F	D6-19*01 F	J1*01 F	CARVQFYWSGSDLRVVGFQHW	V3-19*01 F	J2*01 F, or J3*01 F	CNSRDRSGNYVVF	This work
R3WT_2B2	V1-18*01 F	D6-19*01 F	J1*01 F or J4*02	CARVQLYWSGSDLRVVGFQHW	V1-40*01 F, or V1-40*03 F or V1-50*01 ORF	V1-18*01 F	CAAWDDNLRSHVF	This work

Supplementary Figure S3. An immunoglobulin gene analysis of scFv-derived antibodies in this study.

Supplementary Figure S4

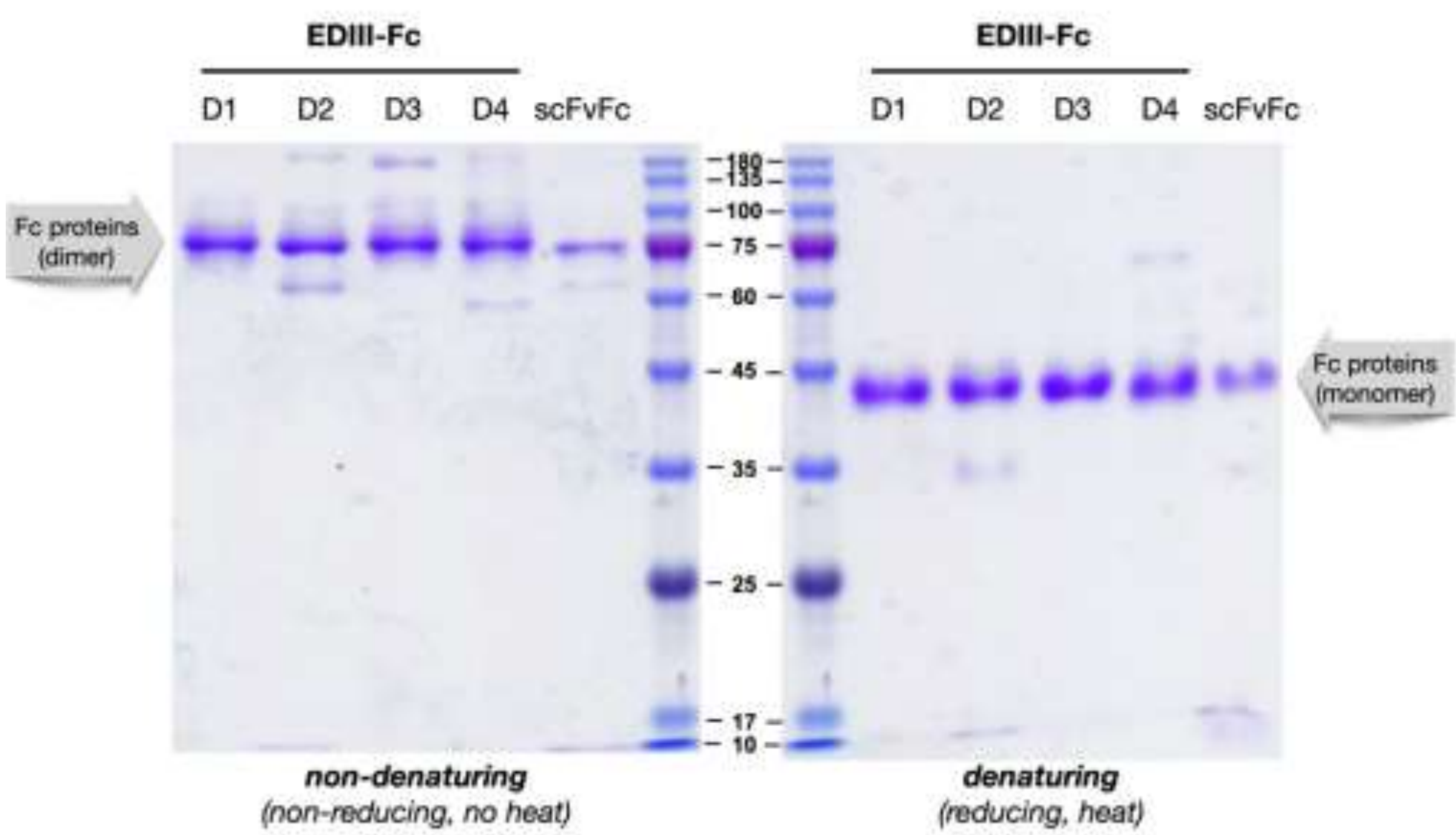

Supplementary Figure S4. Coomassie-stained SDS-PAGE analysis of recombinantly expressed EDIII-Fc antigens and an unrelated Fc-fusion protein (scFvFc) used in ELISA.

Supplementary Figure S5

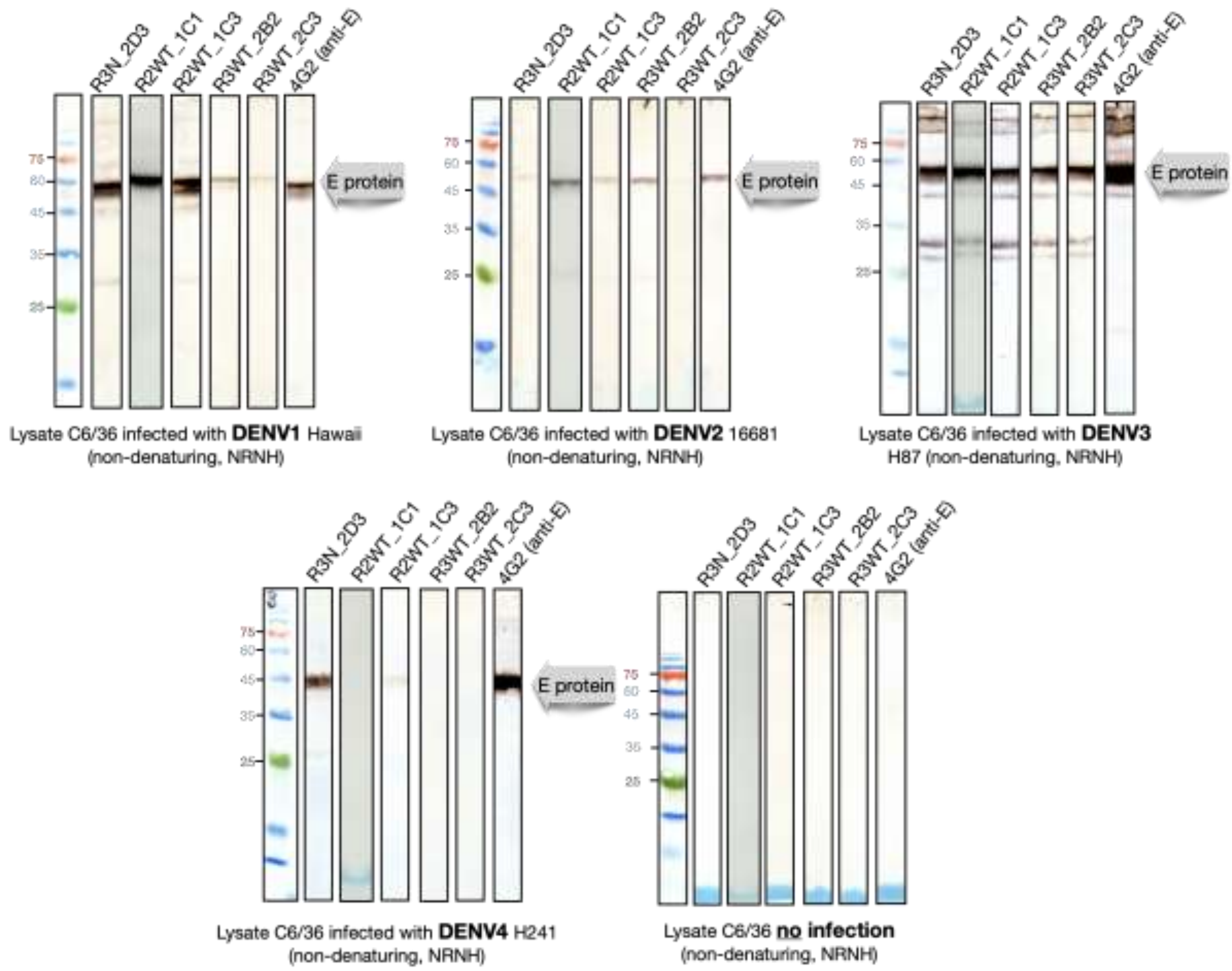

Supplementary Figure S5. Confirmation of DENV envelope (E) protein specificity of antibodies in this study with western blot analysis of DENV-infected C3/36 cell lysates.

Supplementary Figure S6

SPR sensogram: R3N_2D3 – DENV-1 E80

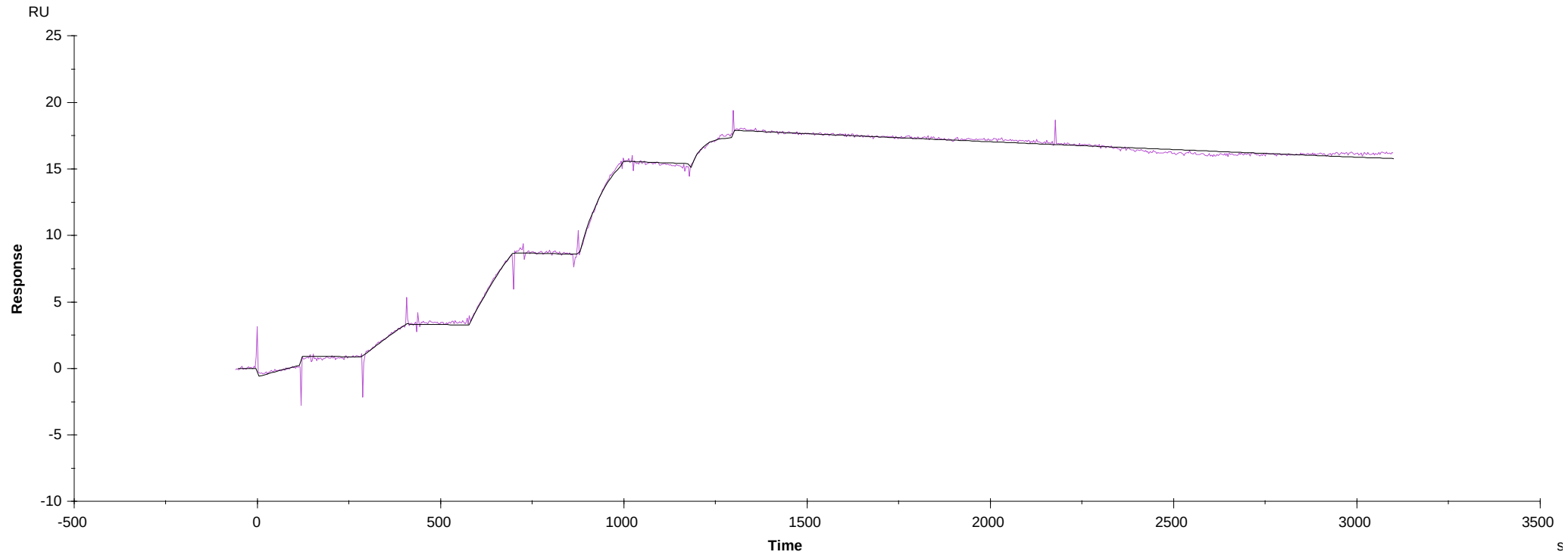

SPR sensogram: R3N_2D3 – DENV-2 E80

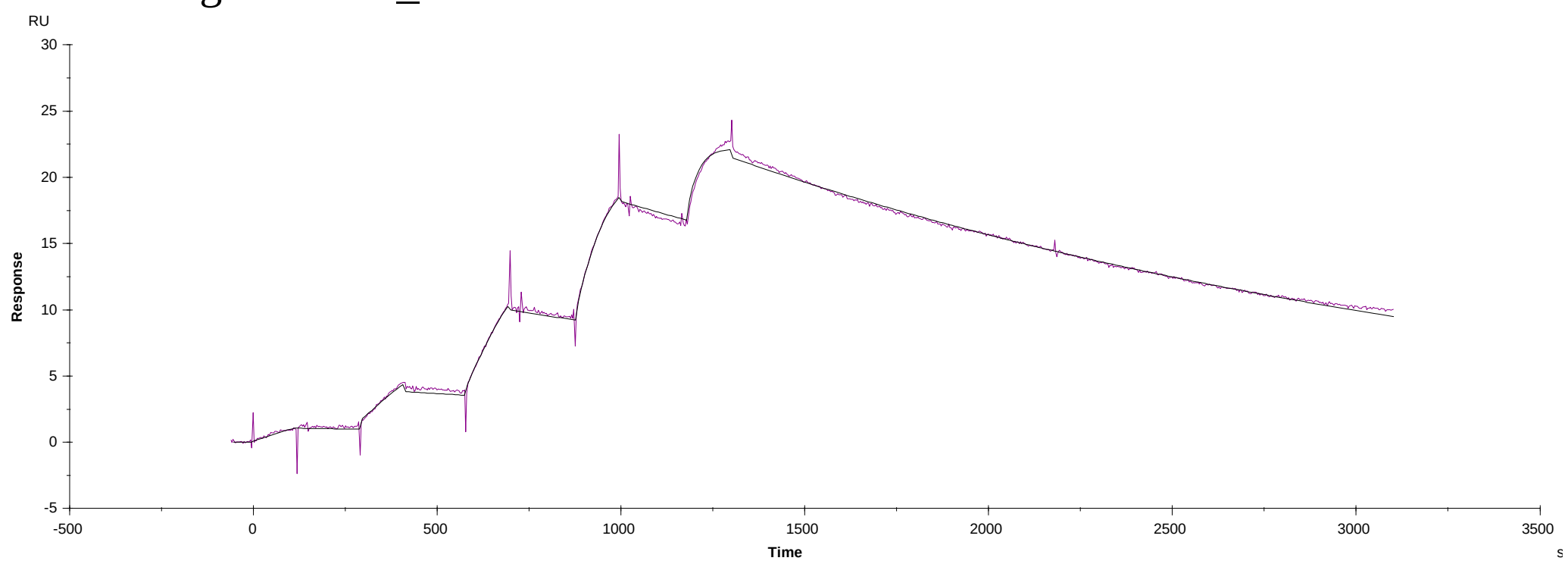

SPR sensogram: R3N_2D3 – DENV-3 E80

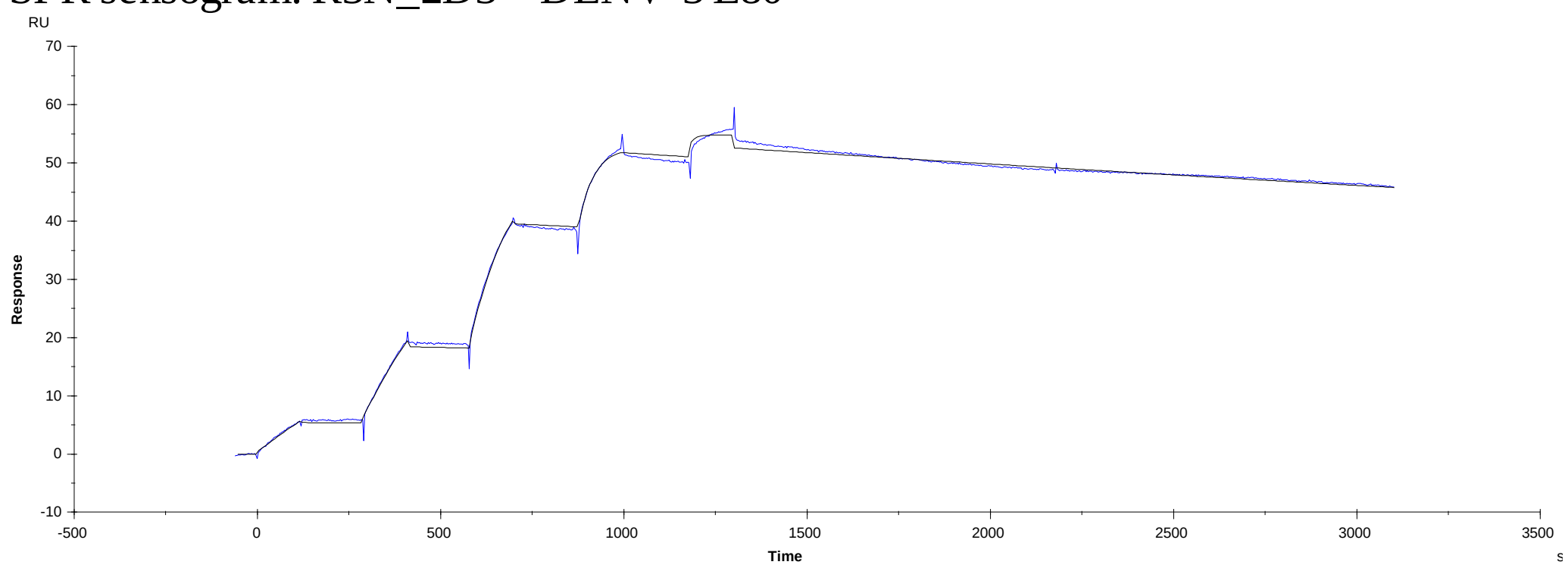

SPR sensogram: R3N_2D3 – DENV-4E80

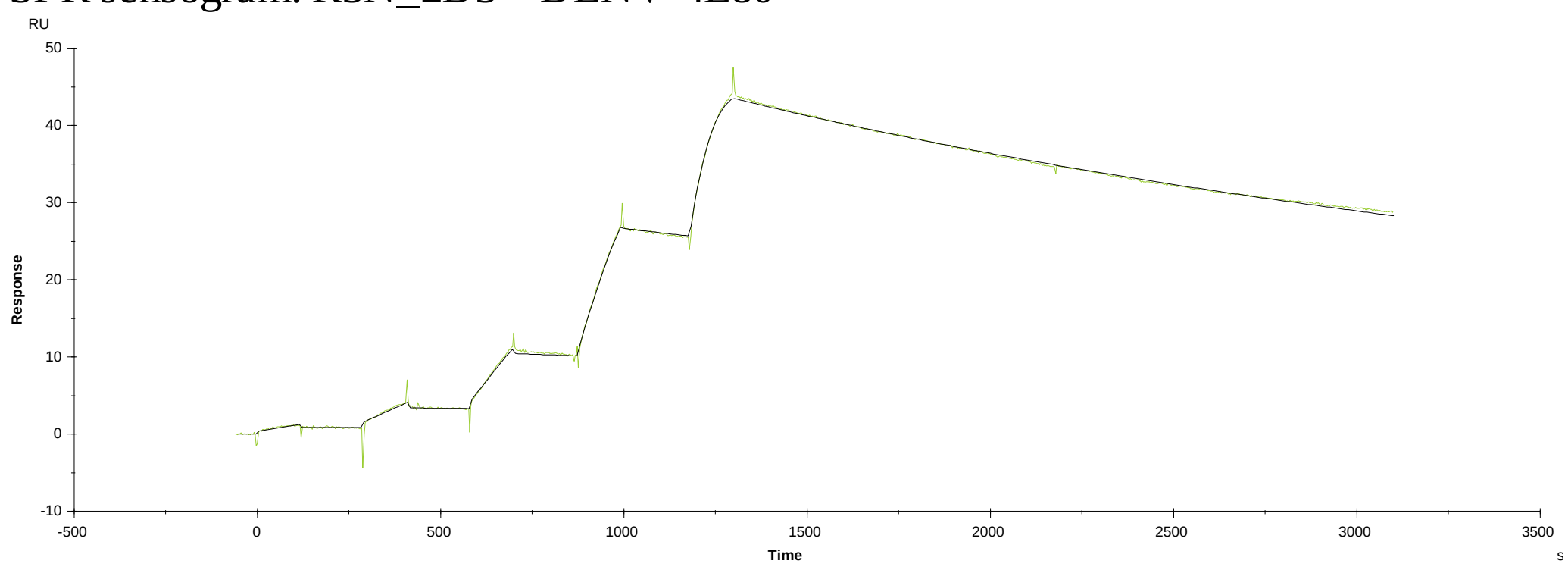

Supplementary Figure S6 (cont.)

SPR sensogram: R2WT _1C1 – DENV-1 E80 (MCK method was employed when SCK gave suboptimal results)

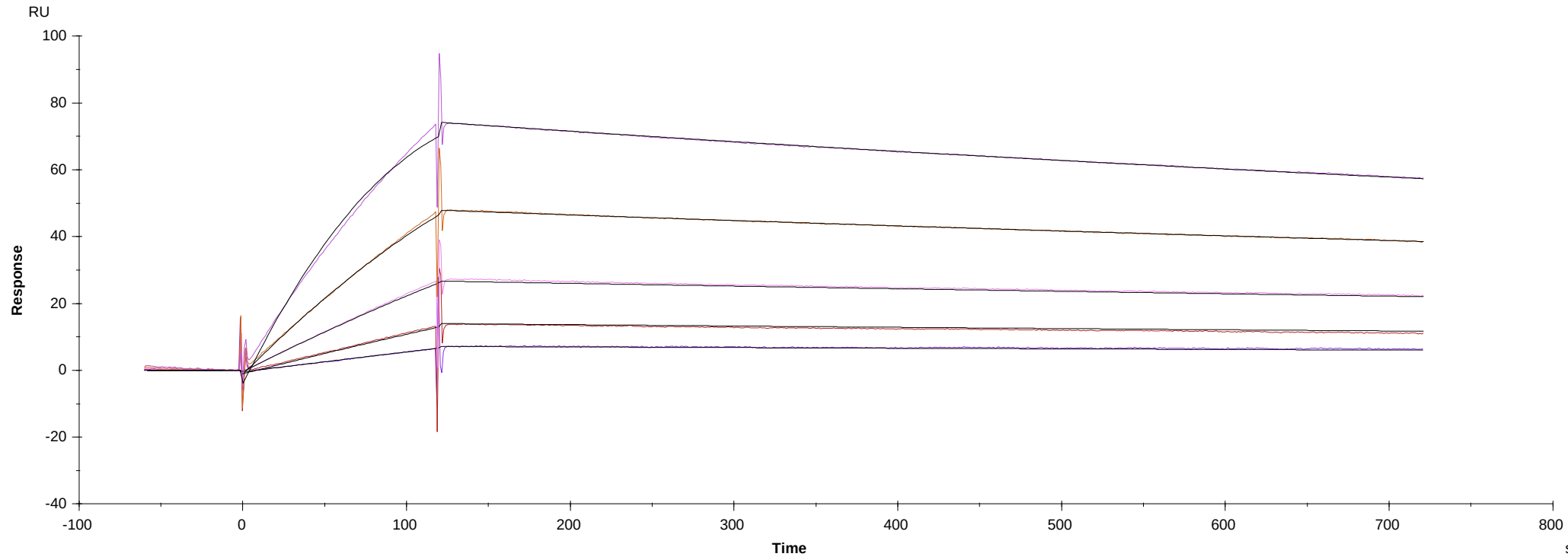

SPR sensogram: R2WT _1C1 – DENV-2 E80 (MCK method was employed when SCK gave suboptimal results)

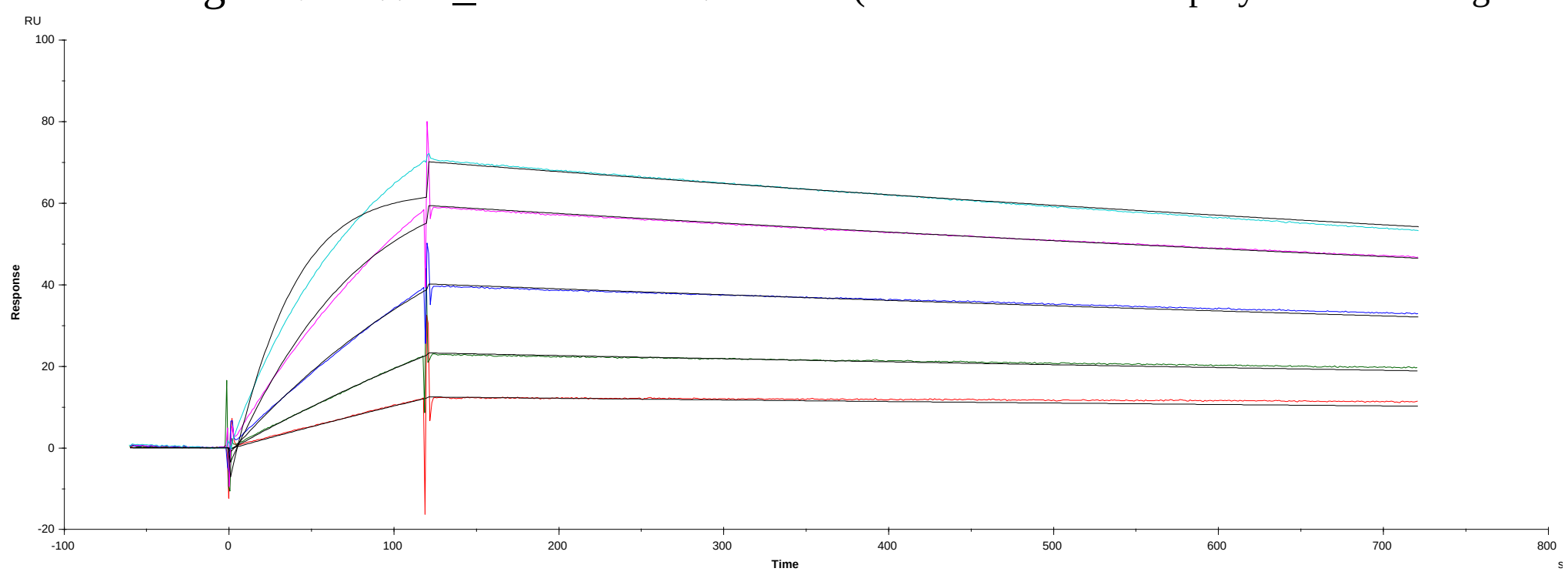

SPR sensogram: R2WT _1C1 – DENV-3 E80

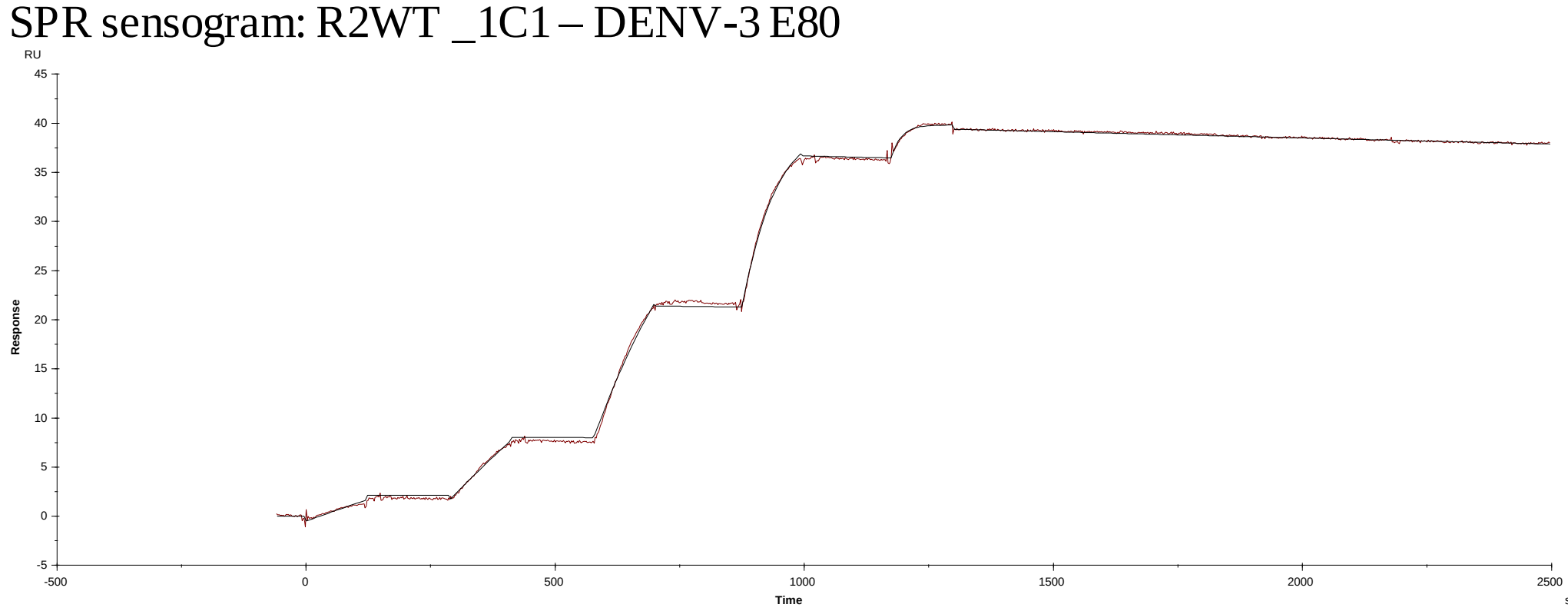

SPR sensogram: R2WT _1C1 – DENV-4E80

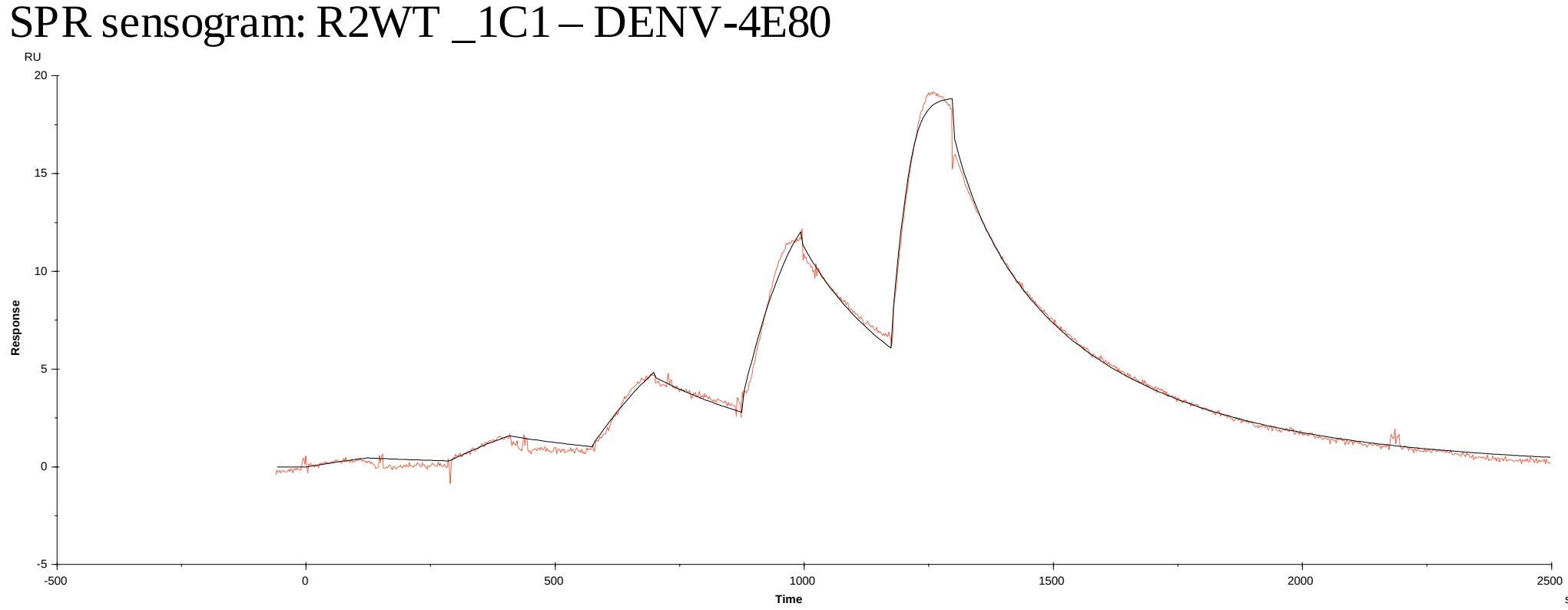

Supplementary Figure S6 (cont.)

SPR sensogram: R2WT _1C3 – DENV-1 E80

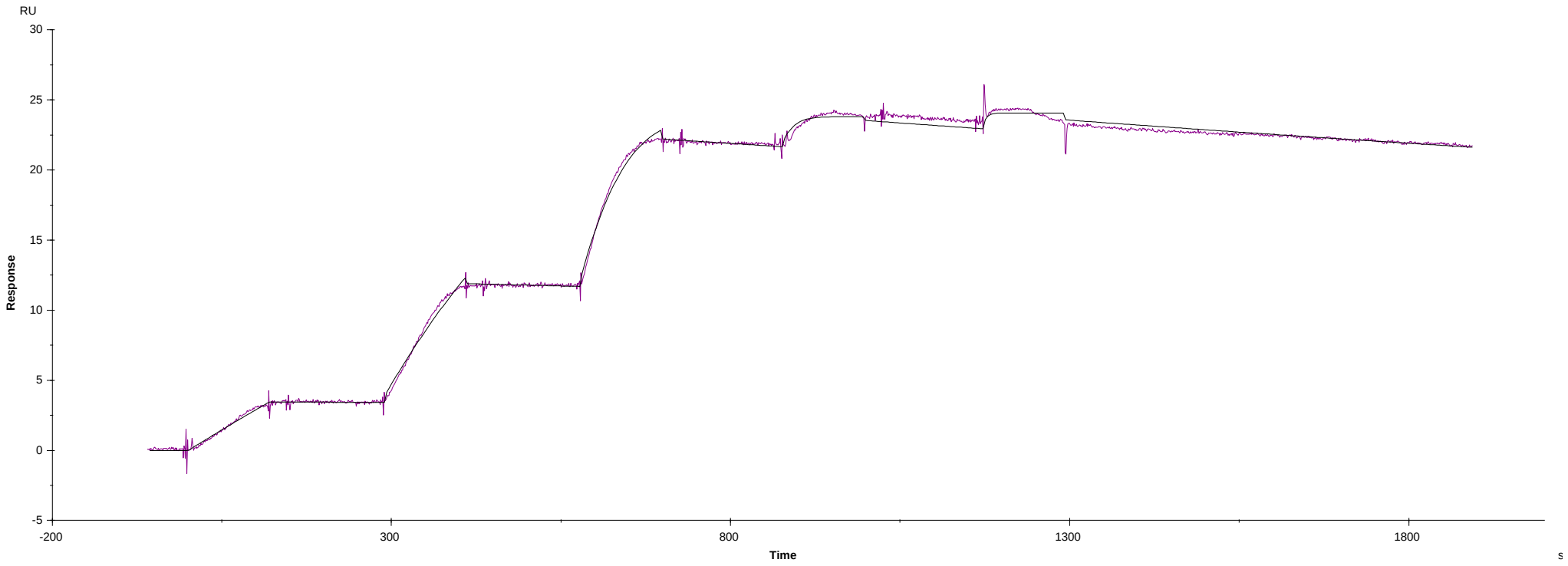

SPR sensogram: R2WT _1C3 – DENV-2 E80

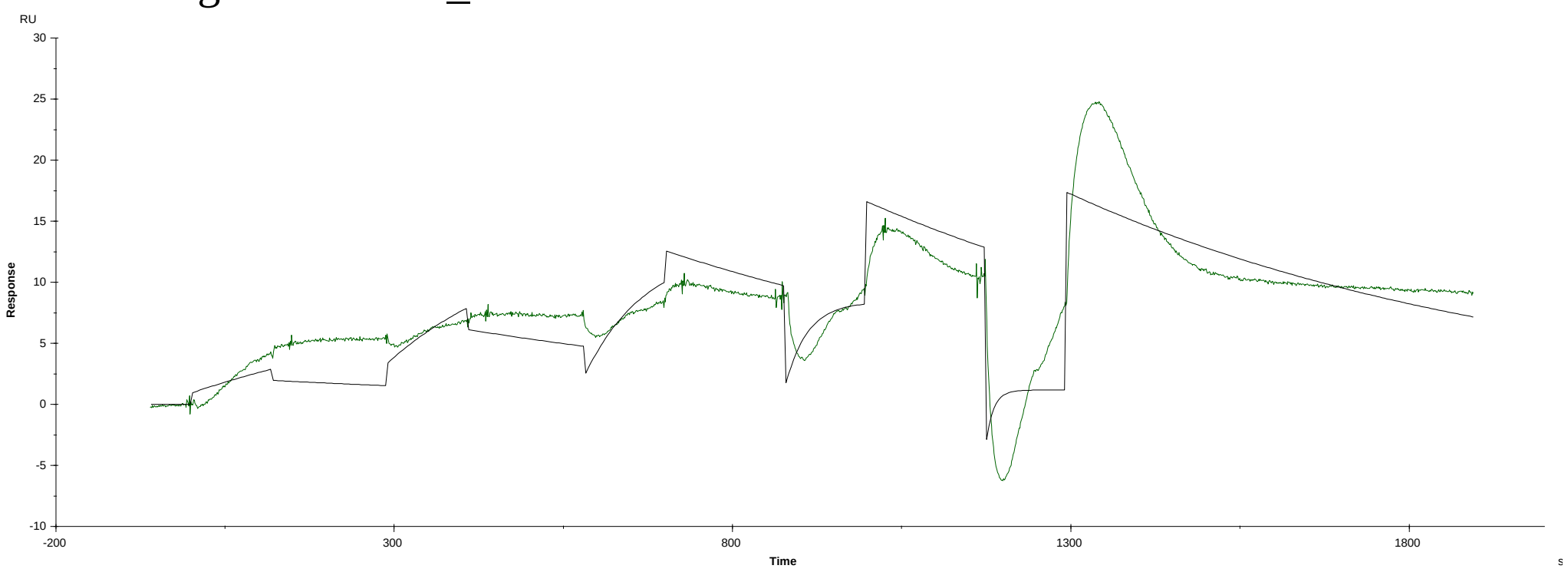

SPR sensogram: R2WT _1C3 – DENV-3 E80

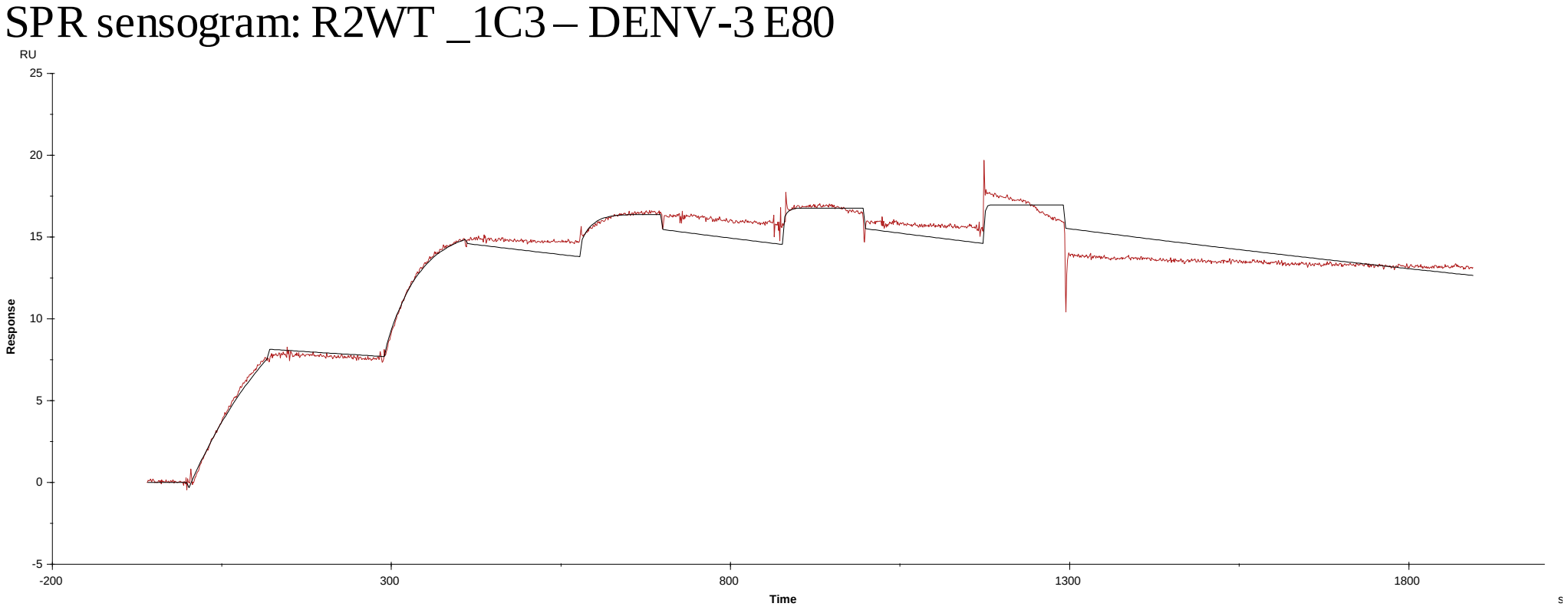

SPR sensogram: R2WT _1C3 – DENV-4E80

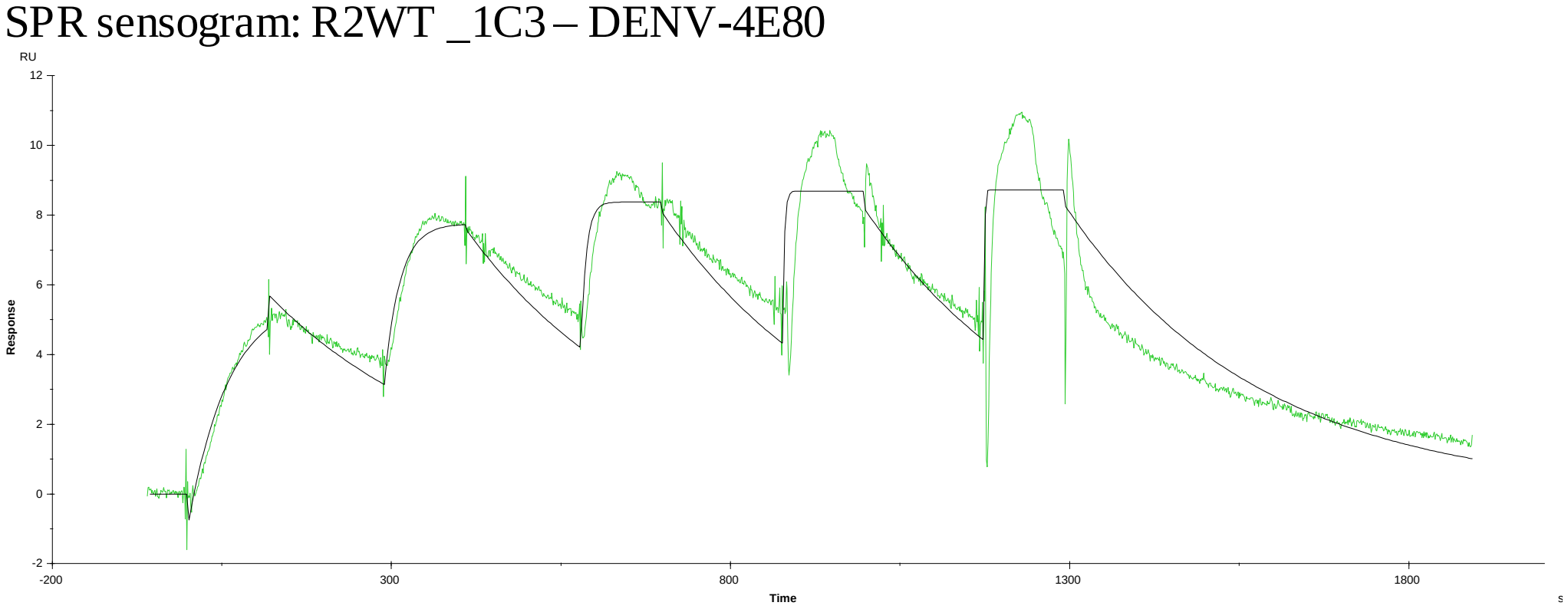

Supplementary Figure S6 (cont.)

SPR sensogram: R3WT_2B2 – DENV-1 E80 (MCK method was employed when SCK gave suboptimal results)

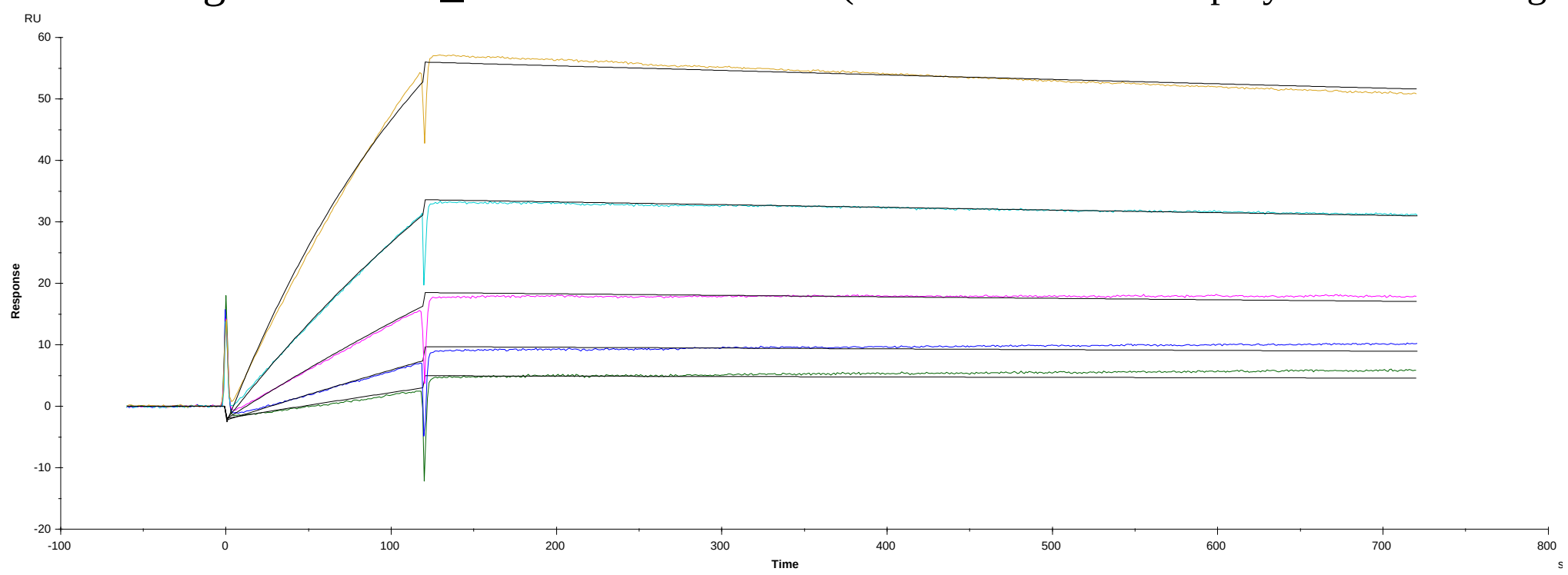

SPR sensogram: R3WT_2B2 – DENV-2 E80 (MCK method was employed when SCK gave suboptimal results)

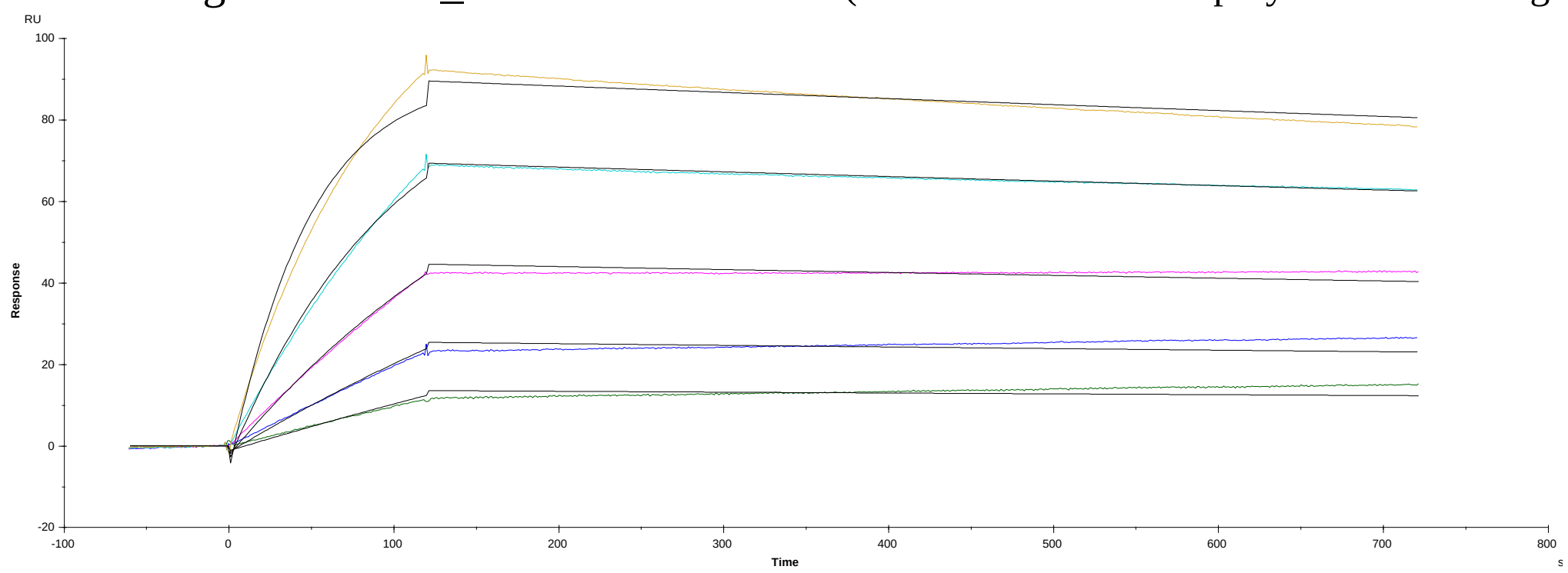

SPR sensogram: R3WT_2B2 – DENV-3 E80

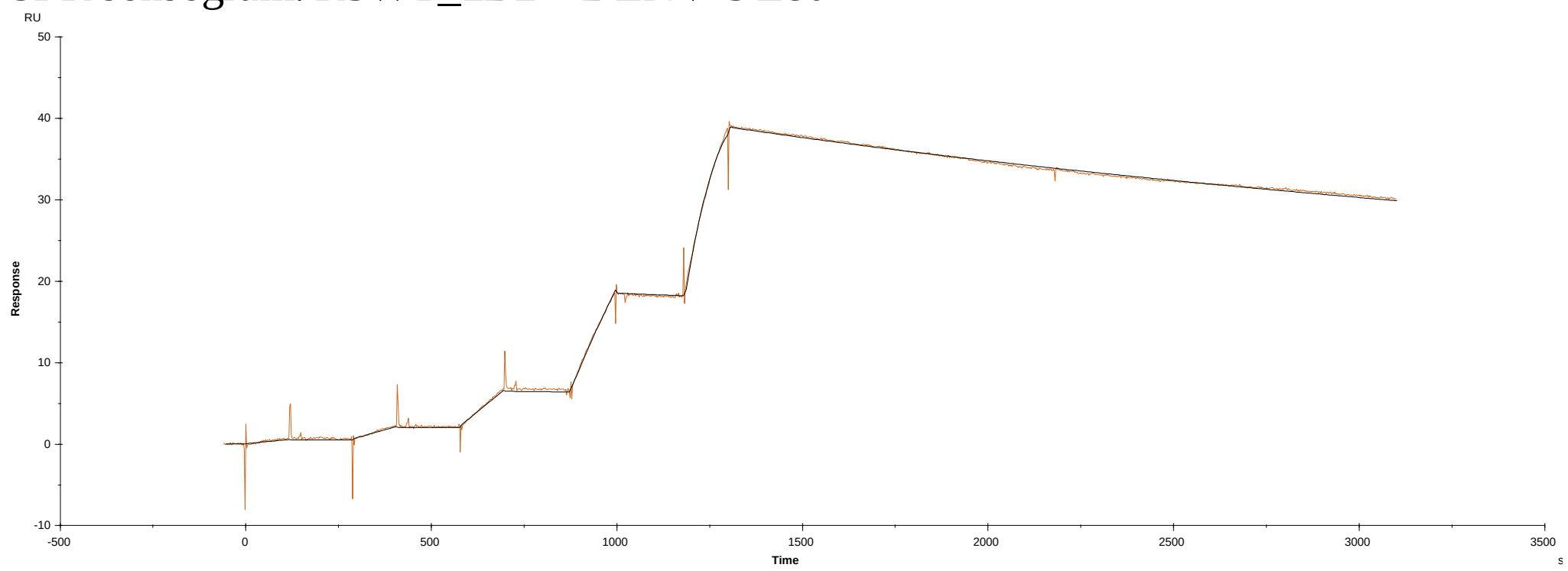

SPR sensogram: R3WT_2B2 – DENV-4E80

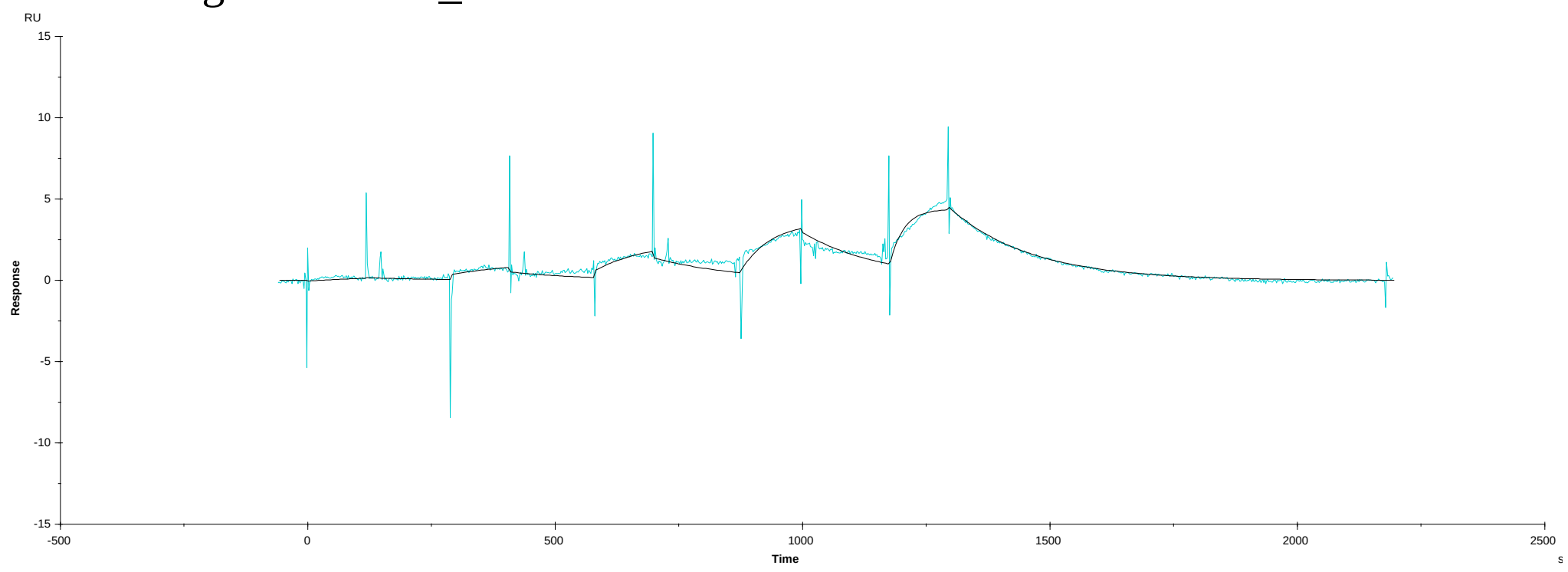

Supplementary Figure S6 (cont.)

SPR sensogram: R3WT_2C3 – DENV-1 E80

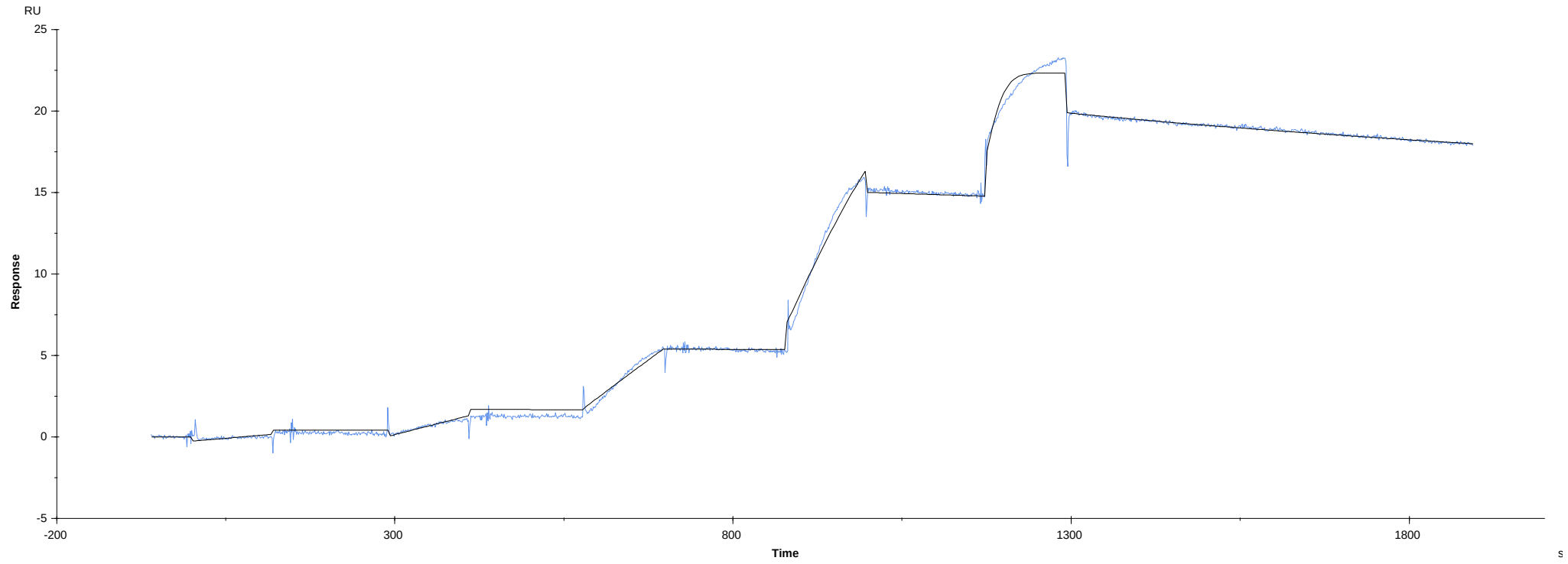

SPR sensogram: R3WT_2C3 – DENV-2 E80

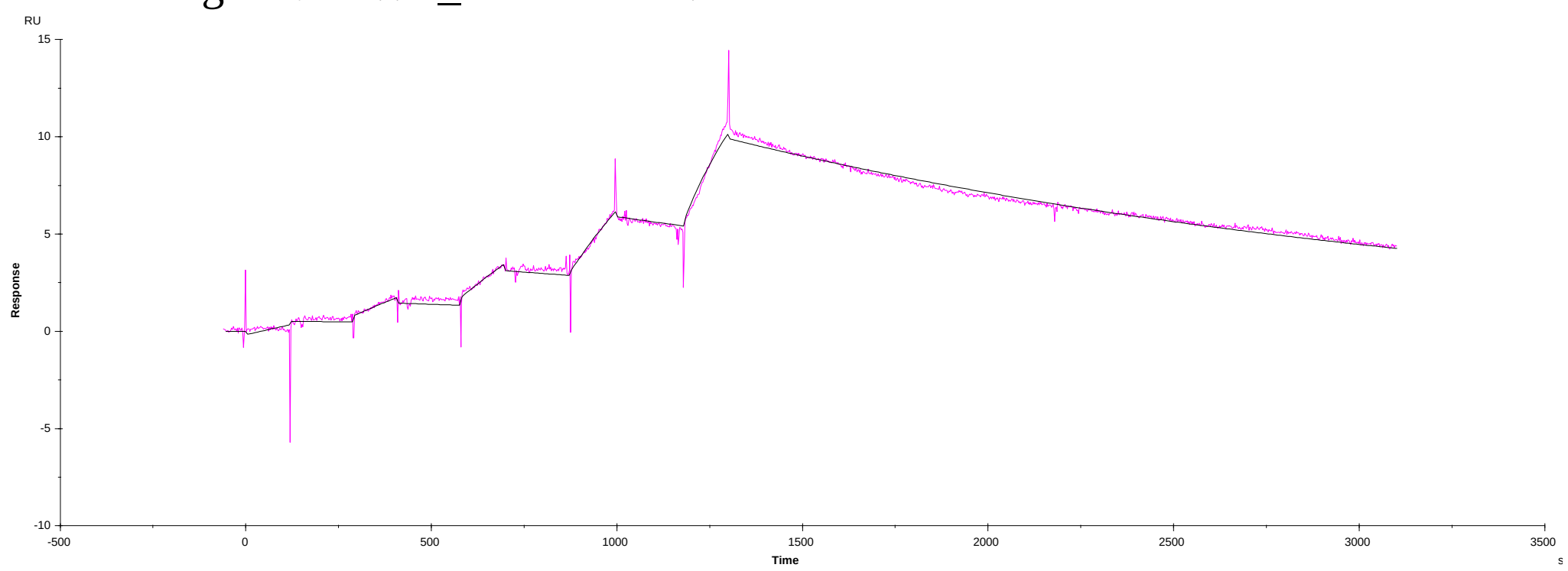

SPR sensogram: R3WT_2C3 – DENV-3 E80

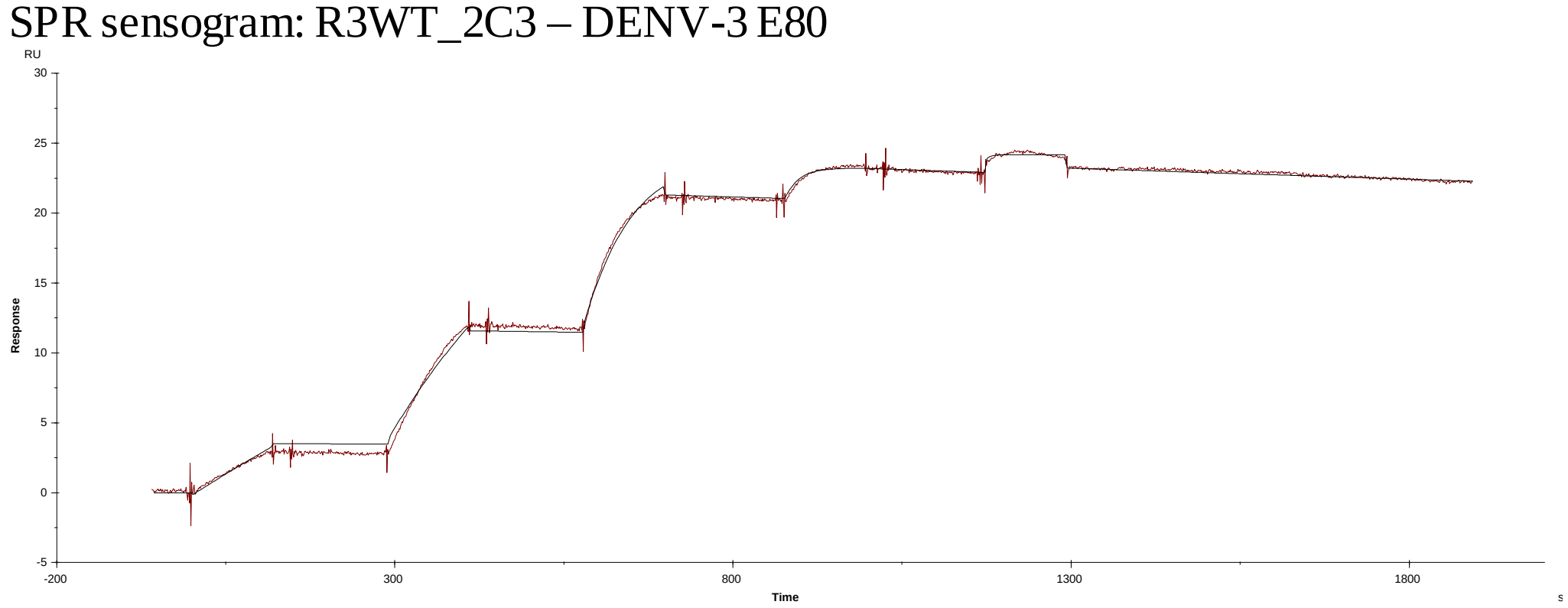

SPR sensogram: R3WT_2C3 – DENV-4E80

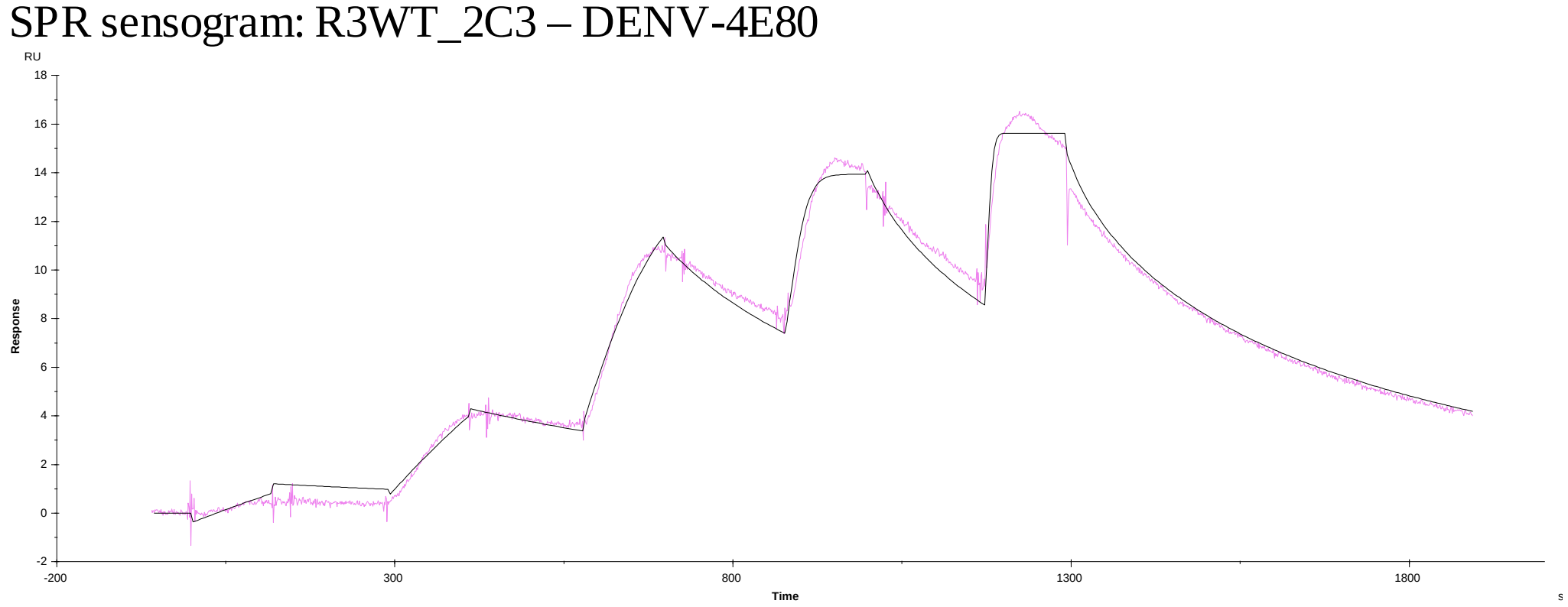

Supplementary Figure S6 (cont.)

SPR sensogram: R2WT_1B8 – DENV-1 E80

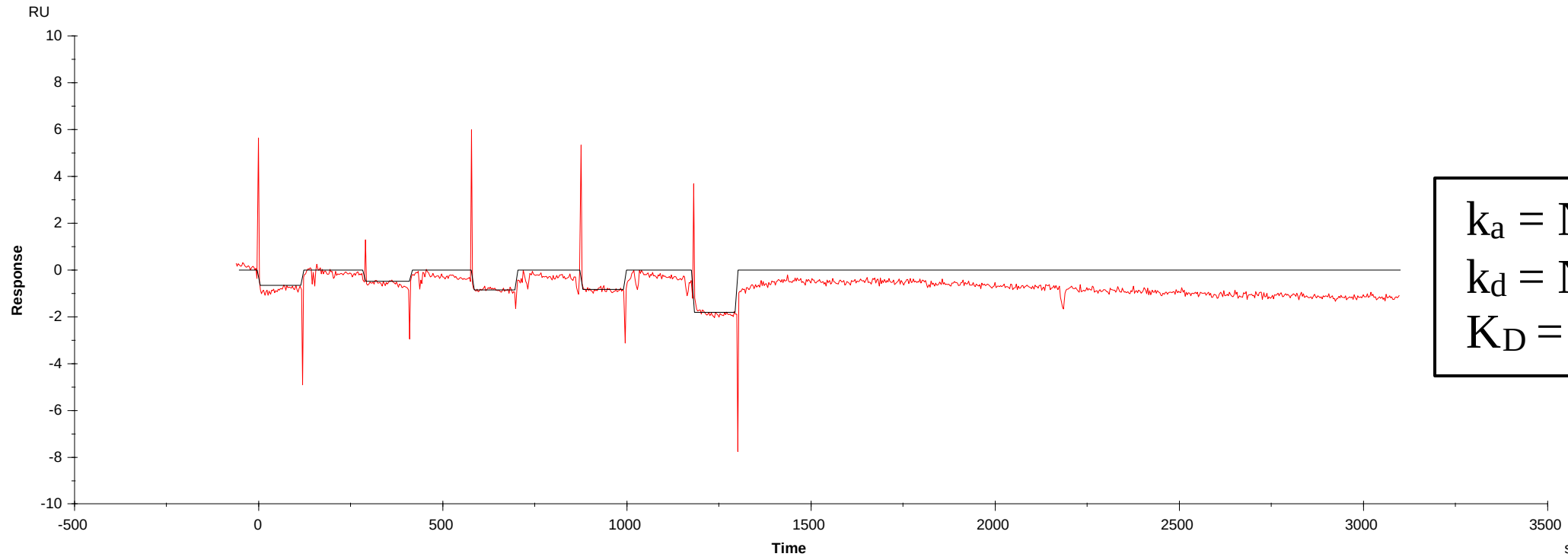

k_a = ND
k_d = ND
K_D = ND

SPR sensogram: R2WT _1B8 – DENV-2 E80

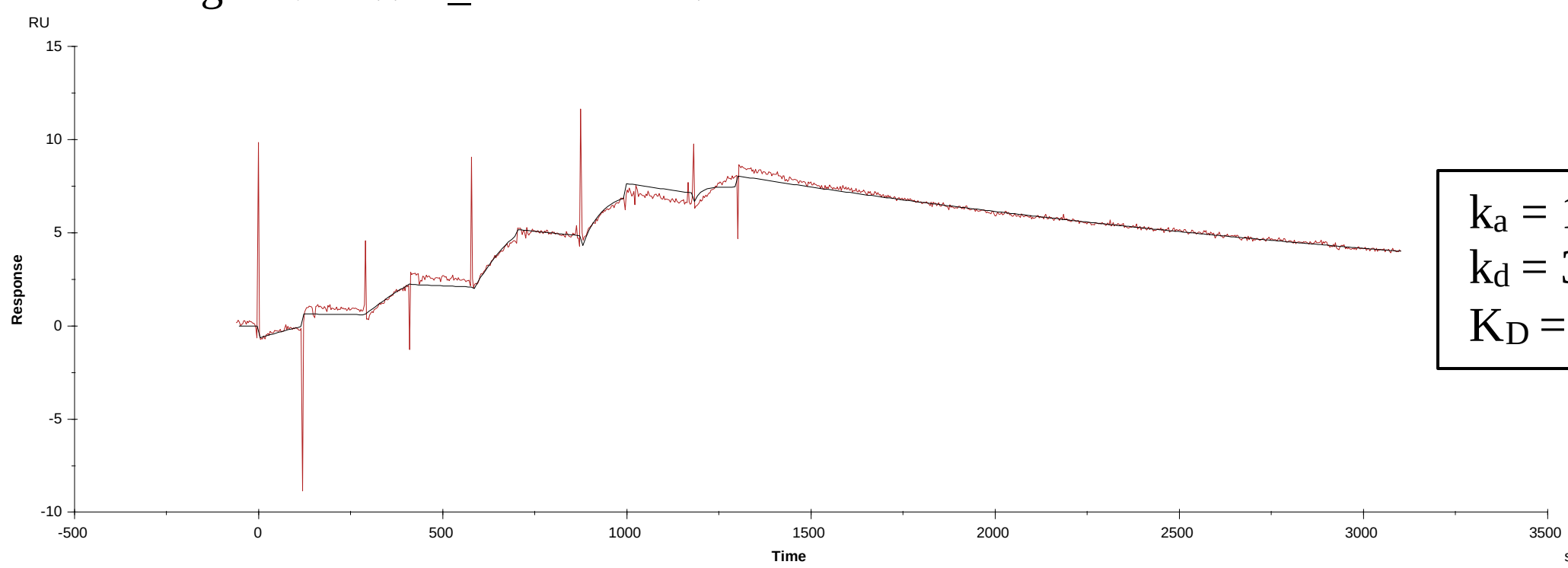

$k_a = 1.15 \times 10^{6}\ M^{-1}s^{-1}$
$k_d = 3.87 \times 10^{-4}\ s^{-1}$
$K_D = 3.37 \times 10^{-10}\ M$

SPR sensogram: R2WT _1B8 – DENV-3 E80

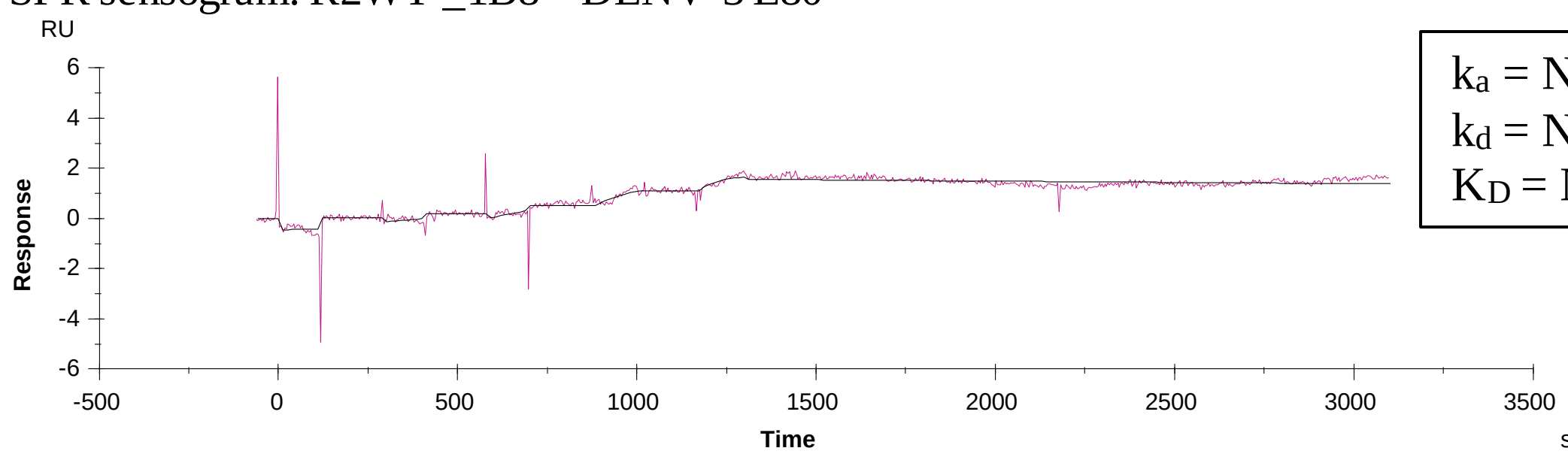

k_a = ND
k_d = ND
K_D = ND

SPR sensogram: R2WT _1B8 – DENV-4E80

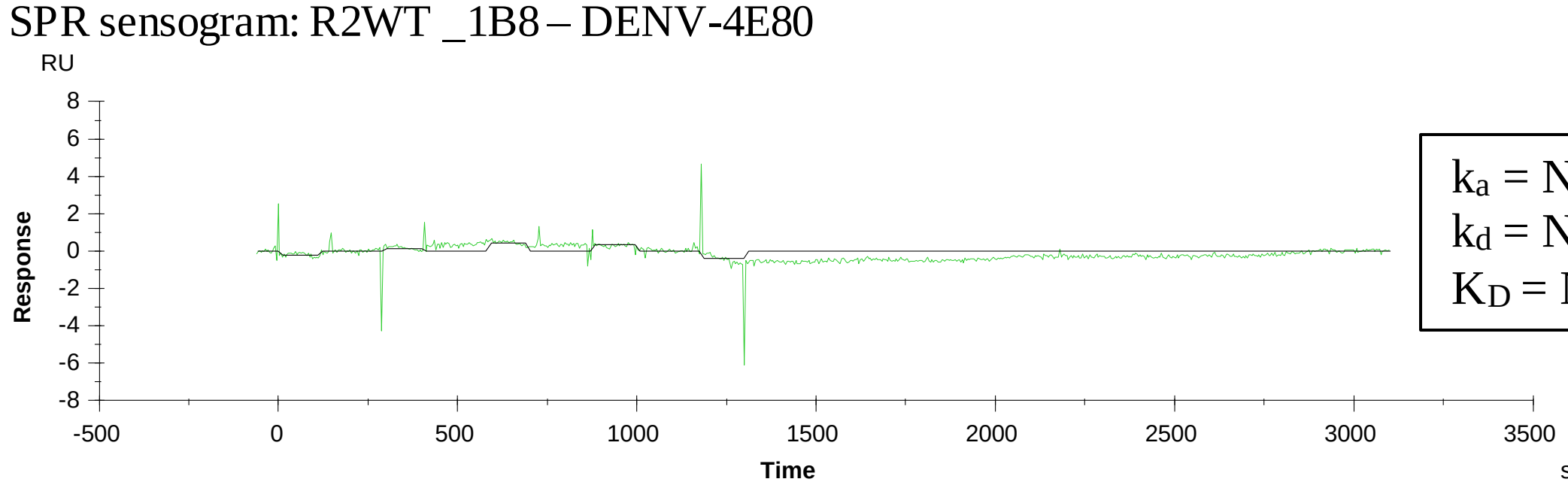

k_a = ND
k_d = ND
K_D = ND

ND = not determined

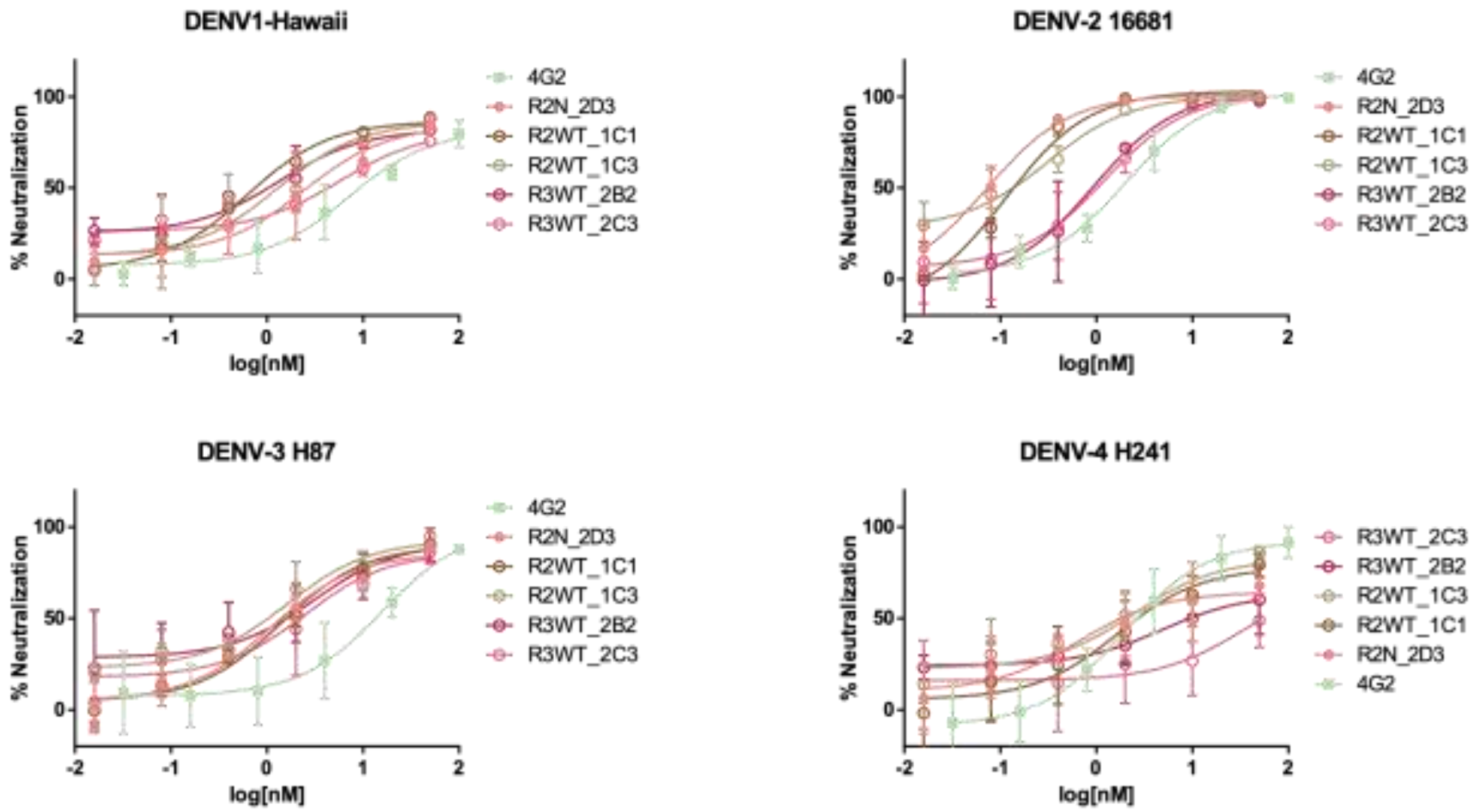

Antibody	FRNT50 (nM)			
	DENV-1 (Hawaii)	DENV-2 (16681)	DENV-3 (H87)	DENV-4 (H241)
R2WT_1C1	0.61	0.11	1.39	1.69
R2WT_1C3	1.27	0.31	1.62	2.46
R2WT_2B2	1.34	0.92	3.09	4.86
R2WT_2C3	5.71	1.22	2.48	32.91
R3N_2D3	2.43	0.07	1.11	0.65
4G2	7.04	2.01	16.2	1.91

Supplementary Figure S7. Focus reduction neutralization titer plots and $FRNT_{50}$ values of cross-reactive AG-strand targeting scFv-derived antibodies in this study.
